# Multi-omics reveals goose fatty liver formation from metabolic reprogramming

**DOI:** 10.3389/fvets.2024.1122904

**Published:** 2024-01-29

**Authors:** Rongxue Wei, Yongqiang Teng, Chunchun Han, Shouhai Wei, Liang Li, Hehe Liu, Shenqiang Hu, Bo Kang, Hengyong Xu

**Affiliations:** ^1^Farm Animal Genetic Resources Exploration and Innovation Key Laboratory of Sichuan Province, Sichuan Agricultural University, Chengdu, China; ^2^Key Laboratory of Livestock and Poultry Multi-Omics, Ministry of Agriculture and Rural Affairs, College of Animal Science and Technology, Sichuan Agricultural University, Chengdu, China

**Keywords:** goose fatty liver, transcriptome, lipidome, amino acid metabolome, peripheral adipose tissue

## Abstract

To comprehensively provide insight into goose fatty liver formation, we performed an integrative analysis of the liver transcriptome, lipidome, and amino acid metabolome, as well as peripheral adipose tissue transcriptome analysis using samples collected from the overfed geese and normally fed geese. Transcriptome analysis showed that liver metabolism pathways were mainly enriched in glucolipid metabolism, amino acid metabolism, inflammation response, and cell cycle; peripheral adipose tissue and the liver cooperatively regulated liver lipid accumulation during overfeeding. Liver lipidome patterns obviously changed after overfeeding, and 157 different lipids were yielded. In the liver amino acid metabolome, the level of Lys increased after overfeeding. In summary, this is the first study describing goose fatty liver formation from an integrative analysis of transcriptome, lipidome, and amino acid metabolome, which will provide a whole new dimension to understanding the mechanism of goose fatty liver formation.

## Introduction

When the geese or ducks were overfed with a high-energy diet that was rich in carbohydrates, their liver increased in size by 5- to 10-fold in 2 to 3 weeks, which was accompanied by the occurrence of hepatic steatosis. The distinctive genetic characteristic of waterfowl was taken advantage of to produce *foie gras*. The process of *foie gras* production by overfeeding is usually simply called liver fattening, which can undoubtedly lead to metabolic disorders. Physiological changes occur when the organism is under the environmental selection pressure induced by microenvironment and genetic factors; meanwhile, metabolic characteristics undergo adaptive changes under the control of genotype, which is called metabolic reprogramming (1). In the early twentieth century, Warburg Otto first found the metabolic reprogramming in tumor cells, namely, “Warburg Effects” (2). In recent years, some scholars have found that hepatocytes metabolic reprogramming during the phenotypic switching from fatty liver to non-alcoholic steatohepatitis (NASH), which is very similar to the “Warburg Effects” (3). Non-alcoholic fatty liver disease (NAFLD) consists of a spectrum of clinical pathological syndromes. When the phenotypic switch from fatty liver to NASH, which represents a crucial step in the progression of NAFLD from simple steatosis to more advanced stages, it is often accompanied by reprogramming liver metabolism to adapt to a stressful metabolic environment, including sugar metabolism, amino acid metabolism, lipid metabolism, nucleotide metabolism, and microbial metabolism. Mass spectrometry-based lipidomics platforms allow for in-depth analysis of lipid alterations in a number of diseases. Human and animal metabolomics and lipidomics data have been used to generate a lipid signature for NAFLD and NASH (4). Key metabolic pathways in NAFLD and NASH that have been identified by metabolomics and lipidomics approaches could potentially be used as biomarkers for non-invasive diagnostic tests (5). Amino acids are the nutrients consumed daily by animals, which are closely related to the lipid metabolism of the body. There is an imbalance in amino acid metabolism that brings new players in the pathogenesis of NASH to our attention (6). Changes in amino acid metabolism are one of the causes of insulin resistance (7). It is generally considered that the formation of fatty liver in geese is primarily due to the imbalance between lipid synthesis, transport, and fatty acid *β*-oxidation in the liver (8). Overfeeding significantly increased the concentration of glucose, total cholesterol (TC), triglyceride (TG), high-density lipoproteins (HDL), alanine transaminase (ALT), aspartate transaminase (AST), and free fatty acid in goose serum (9). These changes in the liver and serum of overfed geese are similar to those in humans with NAFLD. Therefore, waterfowl is the model animal in biomedical research for non-alcoholic fatty liver (10). Some research studies have been carried out to reveal the mechanism of goose fatty liver formation. Previous studies mainly focused on the “gut-liver axis,” gene expression, and nutrition level (11–13). However, to date, the goose fatty liver formation has not been elucidated from the perspective of metabolic reprogramming.

Metabolic reprogramming is an important mechanism for cell survival (14). Of note, geese are the offspring of migratory birds; after energy consumption of stored lipids, their livers are restored to their original state. The entire process was found to be reversible. In addition, neither cirrhosis nor necrosis was observed in the liver (15), suggesting that waterfowl have a mechanism to protect their livers from harm caused by severe hepatic steatosis. This protective mechanism makes migratory birds tolerant of a large amount of lipids deposited in the liver, which provides energy for its long-distance migration. As the goose liver capacity for and tolerance to severe hepatic steatosis, gut microbiota, adiponectin, and liver fatty acid composition play important roles (13, 16, 17). However, the mechanisms underlying the excellent capacity and tolerance of goose liver to severe steatosis still need further exploration. As said above, overfeeding can undoubtedly lead to lipid accumulation and metabolic disorders in overfed goose liver, and metabolic reprogramming is an important mechanism for cell survival. Therefore, metabolic reprogramming will reveal new insight into the existence of a protective mechanism that contributes to the inhibition of inflammation induced by lipid accumulation in goose fatty liver.

In addition, the interaction between peripheral adipose tissue and the liver plays a role in the development of NAFLD (18, 19). A study reported that pigs are protected against fructose-induced steatosis by relying on adipose tissue rather than the liver for *de novo* lipogenesis (20). In mammals, the main site of lipid synthesis is adipose tissue, whereas in geese lipid synthesis is mainly in the liver; the adipose tissue of mammals is the main place for lipid storage, while the liver and the adipose tissue are both important places for fat storage in geese. However, the role of interaction between peripheral adipose tissue and the liver in the development of goose fatty liver is rarely investigated. This current study is made of metabolic reprogramming, which involves in lipidome and amino metabolome after overfeeding, and an integrative analysis was conducted of goose liver transcriptomics, lipidomics, and amino metabolomics. In addition, the interaction between peripheral adipose tissue and the liver during goose fatty liver formation was explored from the transcriptome. These analysis results will provide insight into understanding the mechanisms of goose fatty liver formation. Not only will understanding these mechanisms associated with goose hepatic steatosis provide ideas for the waterfowl breeding selection for *foie gras*, but it is also conducive to improving the production efficiency and quality of *foie gras*. Meanwhile, it will provide not only a scientific basis to ensure animal welfare but also an approach to the prevention and treatment of fatty liver disease in humans.

## Methods and materials

### Ethics statement

All procedures in the present study were subject to approval by the Institutional Animal Care and Use Committee (IACUC) of Sichuan Agricultural University (Permit No. DKY-B20141401), and carried out in accordance with the approved guidelines. All efforts were made to minimize the suffering of the animals. The movement of birds was not restricted before the age of 90 days. The experimental geese were killed with an electrolethaler before harvesting their tissue samples.

### Birds and experiment design and sampling

Forty 100 days-old male Tianfu meat geese were randomly separated into a control group and an overfeeding group. The control group contained 10 geese, and the overfeeding group contained 30 geese. Tianfu meat goose is a lean Chinese commercial breed that is an *A. anser* × *A. cygnoides* hybrid. The breed is a composite of 87.5% Landes (*A. anser*) and 12.5% of Sichuan White (*A. cygnoides*). The population used in this study is closed and has been under selection for over 10 generations (21). The geese of the control group were allowed *ad libitum* access to the same feed as the overfeeding group, and the geese of the overfeeding group were overfed with the boiled maize (maize boiled for 5 min, supplemented with 1% plant oil and 1% salt), and given free access to water. During overfeeding, the daily feed intake gradually increased. The daily feed intake reached 800–1,000 g (5 meals a day) in the overfed group on the 6th day. The temperature and relative humidity of the room were maintained at 25°C and 65% to 70%, respectively, until the end of the experiment. After 12 h of fasting, the geese were slaughtered. After slaughter, the liver samples and peripheral adipose tissues (subcutaneous fatty tissue, abdominal fatty tissue, and intestine-mesentery fatty tissue) were collected and weighed immediately. Peripheral adipose tissues were frozen at −80°C for transcriptome sequencing. Each liver was separated into two parts. The first part was frozen at −80°C for transcriptome sequencing, lipidome determination, and amino acid detection. The second part was collected for histology examination: The cross-sections from the middle of liver samples that were preserved in 4% formaldehyde-phosphate buffer were prepared using standard paraffin embedding techniques, sectioned (5 μm) and stained with hematoxylin and eosin (HE), sealed by neutral resin size thereafter, and then examined by microscope photography system (Olympus, Tokyo, Japan); each slice was observed, and five visual fields were randomly selected at 200× magnification.

### Transcriptome sequencing and analysis

We collected liver tissues and peripheral adipose tissues (subcutaneous fatty tissue, abdominal fatty tissue, and intestine-mesentery fatty tissue) for total RNA sequencing. Six geese representing three biological replicates for each group (the control group and the overfeeding group) were randomly selected in this experiment. Total RNA from frozen liver tissues (approximately 100 mg) was extracted using RNeasy Mini Kit (QIAGEN, Germany) following the manufacturer’s instructions. RNA integrity was checked by Agilent Bioanalyzer 2100 (Agilent Technologies, CA, United States). Sequencing libraries were generated using the NEBNext UltraTM RNA Library Prep Kit for Illumina NexSeq500 (NEB, United States) following the manufacturer’s recommendations, and index codes were added to attribute sequences to each sample. All libraries were sequenced by the Illumina NexSeq500 platform, which was performed by Suzhou PANOMIX Biomedical Tech Co., Ltd. (Jiangsu, China). After removing sequencing adaptors, duplicated sequences, and low-quality reads from raw data, we obtained high-quality reads, which are called clean reads. Clean reads were mapped against the goose reference genome of geese (*A. cygnoides*) reference genome (assembly Ans Cyg_PRJNA183603_ v1.0, https://www.ncbi.nlm.nih.gov/genome/31397?genome_assembly_id=229313). Splicing information from the reference annotation file (GTF file) was used to guide the RNA-seq reads mapping via Tophat2. The numbers of gene reads were normalized by reads per kilobase of transcript per million fragments mapped (RPKM). Differentially expressed tags between the two groups were identified using DEGseq software. The resulting *p*-values were adjusted using Benjamini and Hochberg’s approach for controlling the false discovery rate. Genes with an adjusted *p*-value less than 0.05 found by DESeq were assigned as differentially expressed genes. Transcriptome analysis was performed via Suzhou PANOMIX Biomedical Tech Co., Ltd. (Jiangsu, China).

### Determination of liver lipidome

Six liver samples that were collected from the control group and six liver samples that were collected from the overfeeding group were sent for lipidome detection. Lipid extraction was performed as follows: (1) 100 mg of each sample was transferred into 2 mL centrifuge tubes, and 750 μL of chloroform/methanol mixed solution (2:1) (pre-cooled at −20°C) and two steel balls (the insufficient sample size is reduced to an equal scale) were added; (2) the samples were ground by a high flux organization grinding apparatus for 60 s at 60 Hz; (3) the samples were put on the ice for 40 min, 190 μL L ddH_2_O was added and vortex mixed for 30 s, and put on the ice for 10 min; (4) the samples were centrifuged at 12,000 rpm for 5 min at room temperature and 300 μL of lower layer fluid was transferred into a new centrifuge tube; (5) 500 μL of chloroform/methanol mixed solution (2:1) (pre-cooled at −20°C) was added and vortex mixed for 30 s; (6) the samples were centrifuged at 12,000 rpm for 5 min at room temperature and 400 μL of lower layer fluid was transferred into the same centrifuge tube mentioned above. Samples were concentrated to dry in a vacuum; (7) samples were dissolved with 200 μL isopropanol, and the supernatant was filtered through 0.22 μm membrane to obtain the prepared samples for LC-MS; (8) 20 μL from each sample was taken to the quality control (QC) samples (these QC samples were used to monitor deviations of the analytical results from these pool mixtures and compare them to the errors caused by the analytical instrument itself); (9) the rest of the samples were used for liquid chromatography-mass spectrometry (LC-MS) detection.

Chromatographic separation was accomplished in a Thermo Vanquish system equipped with an ACQUITY UPLC^®^ BEH C18 (100 × 2.1 mm, 1.7 μm, Waters) column maintained at 50°C. The temperature of the autosampler was 8°C. Gradient elution of analytes was carried out with acetonitrile:water = 60:40 (0.1% formic acid + 10 mM ammonium formate) (A2) and isopropanol:acetonitrile = 90:10 (0.1% formic acid + 10 mM ammonium formate) (B2) at a flow rate of 0.25 mL/min. Injection of 2 μL of each sample was done after equilibration. An increasing linear gradient of solvent A (v/v) was used as follows: 0–5 min, 70–57% A2; 5–5.1 min, 57%–50% A2; 5.1–14 min, 50%–30% A2; 14–14.1 min, 30% A2; 14.1–21 min, 30%–1% A2; 21–24 min, 1% A2; 24–24.1 min, 1%–70% A2; 24.1–28 min, 70% A2.

The ESI-MSn experiments were executed on the Thermo Q Exactive Focus mass spectrometer with the spray voltage of 3.5 kV and −2.5 kV in positive and negative modes, respectively. Sheath gas and auxiliary gas were set at 30 and 10 arbitrary units, respectively. The capillary temperature was 325°C, respectively. The Orbitrap analyzer scanned over a mass range of *m*/*z* 150–2,000 for a full scan at a mass resolution of 35,000. Data-dependent acquisition (DDA) MS/MS experiments were performed with an HCD scan. The normalized collision energy was 30 eV. Dynamic exclusion was implemented to remove some unnecessary information in MS/MS spectra. Lipidome determination and analysis were performed by Suzhou PANOMIX Biomedical Tech Co., Ltd. (Jiangsu, China).

### Determination for free amino acid

After grinding, approximately 0.5 g liver sample was used to mix with 2 mL of 0.01 mol/L hydrochloric acid in a special tube (for homogenization). After homogenization, homogenate was taken into a 15 mL centrifugal tube, and centrifuged at 8,000 × g for 10 min. This process was repeated in triplicate. The upper solution was transferred to a 10 mL volumetric flask and then diluted with pure water and mixed; 800 μL of sample liquid was measured accurately and mixed with 200 μL of 10% salicylic acid in a 2 mL centrifuge tube, refrigerated at 2°C–8°C for 60 min, and then centrifuged at 12,000 × g for 15 min; the upper solution was transferred to another 2 mL centrifuge tube for vacuum drying. After vacuum drying, the dry matter was dissolved by 1 mL of 0.01 mol/L hydrochloric acid and then filtered through a 0.22 μm filter membrane to the sample bottle for determination. Amino acid detection used automated online OPA and FMOC derivatization methods. The method for amino acid determination is shown in Supplementary material. According to the corresponding relationship between retention time (RT) and the order of amino acid peaks, a single amino acid standard was used for qualitative analysis. Seventeen single amino acid standards were obtained from Huaxia Chemistry Co., Ltd. (Chengdu, China). The amino acid level was calculated with the area normalization method.

### Data analysis

Slaughter performance data and amino acid data were expressed by mean ± SD and shown with graphs created with GraphPad Prism software (GraphPad 8.0 Software, Inc.). We considered *p* < 0.05 as statistically significant. KOBAS software was used to test the statistical enrichment of the differentially expressed genes (DEGs) in KEGG pathways. Enrichment analysis for three Gene Ontology (GO) terms (biology process, molecular function, and cellular component) was performed. The LipidSearch software (V4) was applied to study the lipidome profile difference between the control group and the overfed group. Multivariate statistical analysis methods were implemented to analyze the lipidome data. Cluster analysis, correlation analysis, principal component analysis (PCA), partial least squares discriminant analysis (PLS-DA), and orthogonal partial least squares-discriminant analysis (OPLS-DA) were performed. The significantly different lipids were screened from the OPLS-DA model (VIP >1.0 and *p* < 0.05). The heat map of data normalized by *Z*-score was generated using TBtools. Then, the KEGG database was used to search for the related KEGG pathways of the lipids and amino acids. Annotation of lipid species was performed as follows: glycerolipids were referred to as TG, diglyceride (DG), monogalactosyldiacylglycerol (MGDG), and monogalactosylmonoacylglycerol (MGMG); glycerophospholipids and lyso-glycerophospholipids were referred to phosphatidic acids (PA), phosphatidylinositols (PI), phosphatidylcholines (PC), phosphatidylethanolamines (PE), lyso-phosphatidylcholines (LPC), and phosphatidylglycerol (PG); sphingolipids were referred to ceramides (Cer), hexaglycosylceramides (Hex1Cer), and sphingomyelins (SM).

## Results

### Overfeeding influence on liver and peripheral adipose tissues transcriptome

**Figure 1:**
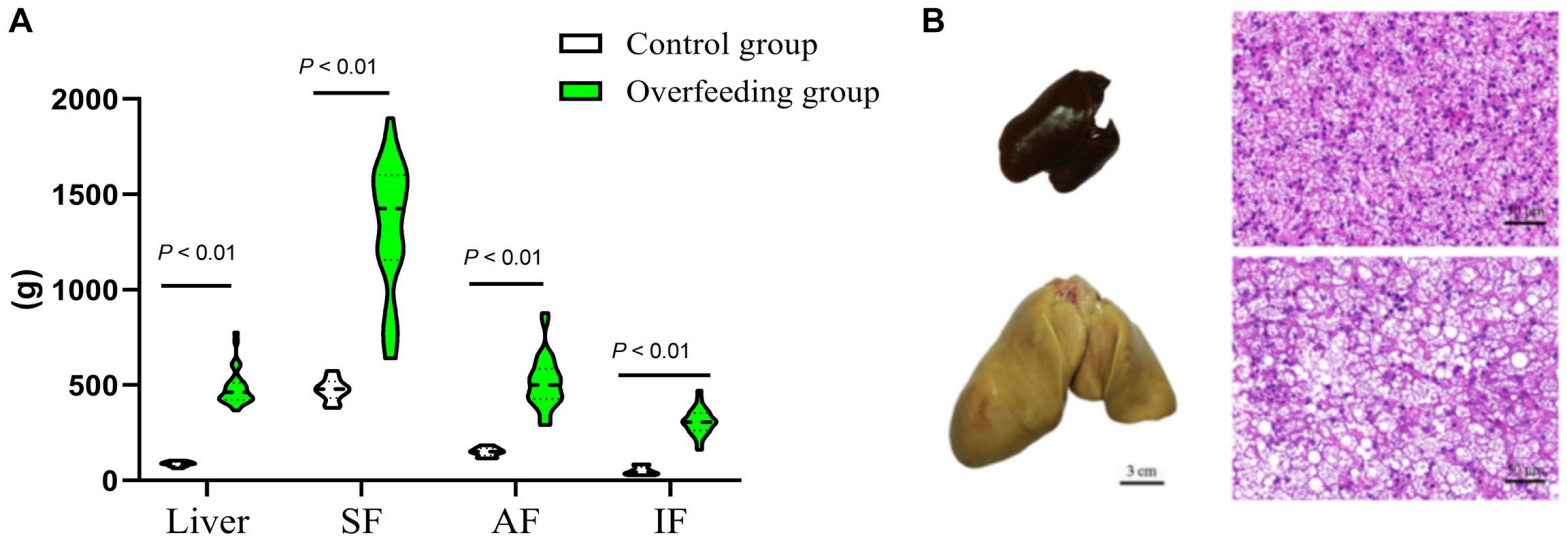
Comparison of livers and peripheral adipose tissue sections between the control group (*n* = 10) and overfed geese (*n* = 30). **(A)** Overfeeding influence on liver weight, subcutaneous fat weight, abdomen fat weight, and intestine-mesentery fat weight. **(B)** Overfeeding-induced hepatic steatosis (liver tissue section 200×). SF, subcutaneous fat; AF, abdominal fat; IF, intestine-mesentery fat.

**Figure 2:**
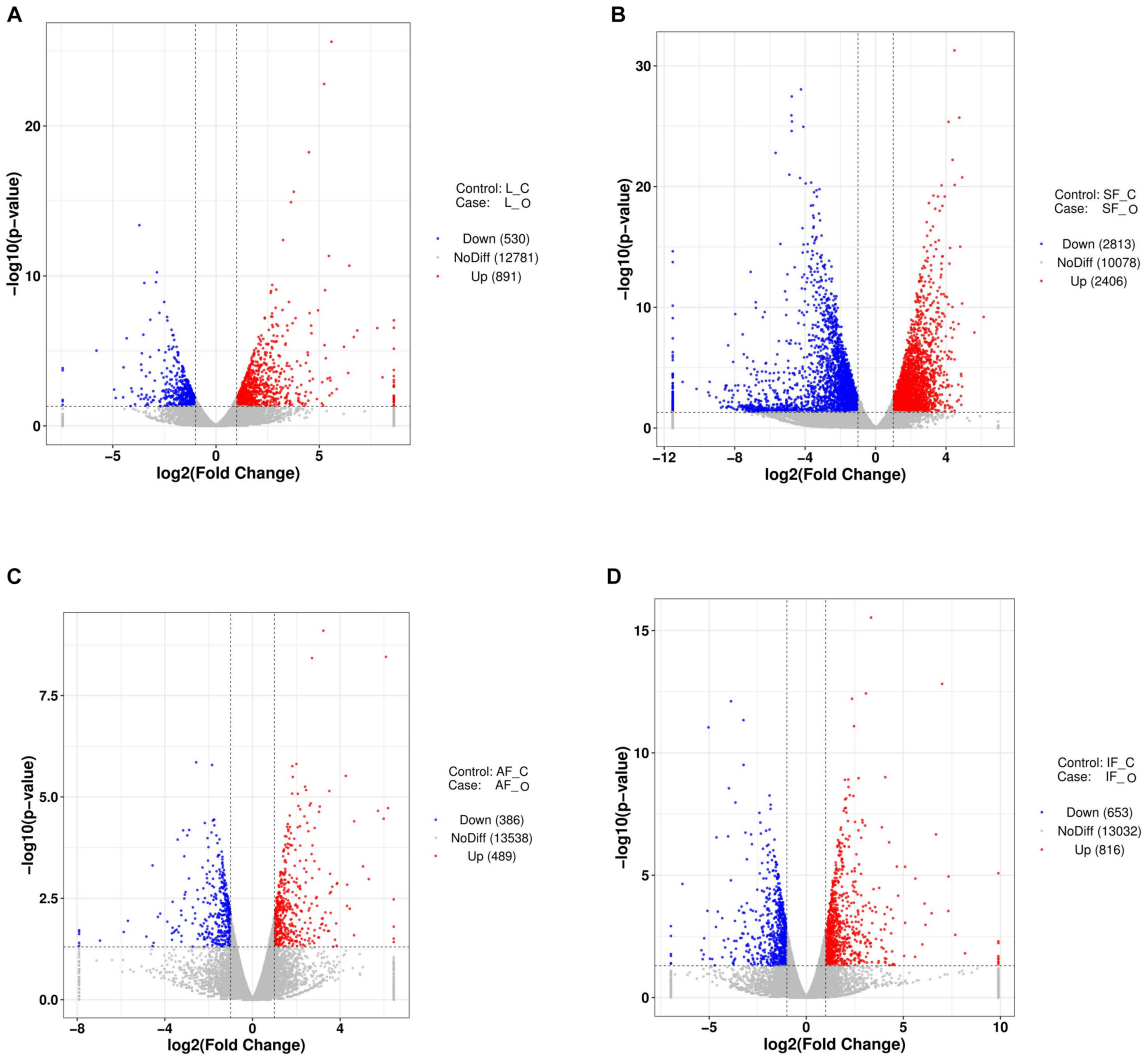
Volcano plots of DEGS from transcriptome analysis (*n* = 3). **(A)** The overfeeding group compared to the control group in liver tissue. **(B)** The overfeeding group compared to the control group in subcutaneous fat tissue. **(C)** The overfeeding group compared to the control group in abdomen fat tissue. **(D)** The overfeeding group compared to the control group in the intestine-mesentery fat tissue. O, overfeeding group; C, control group; SF, subcutaneous fat; AF, abdominal fat; IF, intestine-mesentery fat.

**Figure 3:**
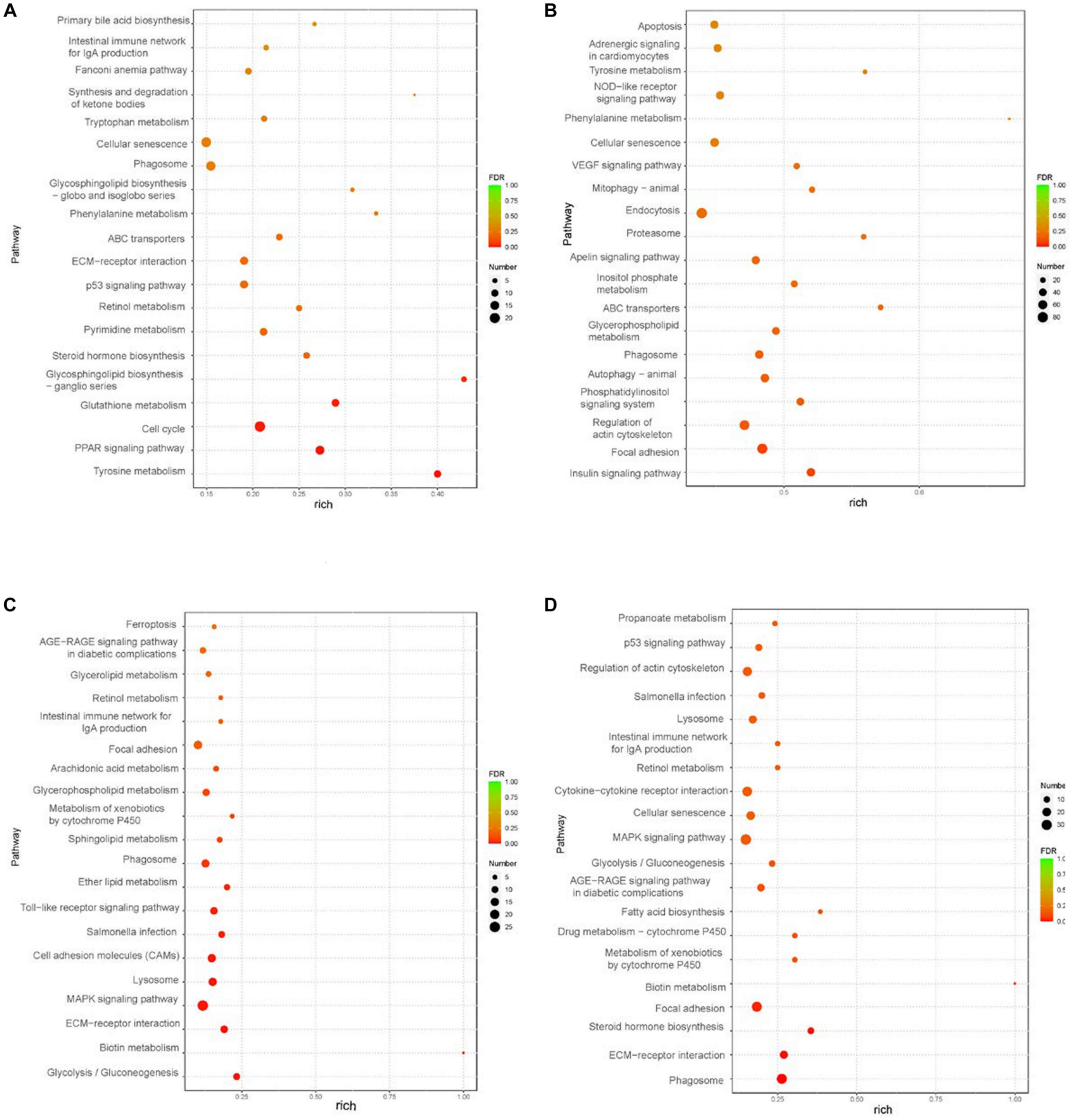
KEGG analysis of liver and peripheral adipose tissue (control group vs. treatment group) (*n* = 3). **(A)** KEGG analysis of liver tissue. **(B)** S2 KEGG analysis of subcutaneous fat tissue. **(C)** S3 KEGG analysis of abdomen fat tissue. **(D)** KEGG analysis of intestine-mesentery fat tissue.

The liver weight, body weight, and peripheral adipose tissue weight (subcutaneous fatty tissue, abdominal fatty tissue, and intestine-mesentery fatty tissue) significantly increased; histology examination showed that severe steatosis was induced in the livers after overfeeding, which suggested that the goose fatty liver model had been successfully built (Figure 1). The liver transcriptome analysis showed that clean reads were mapped to the goose reference genome database, approximately 34.27, 43.78, 35.58, and 34.59 million reads (more than 97%) were mapped uniquely in liver tissue, subcutaneous fat tissue, abdominal fat tissue, and intestine-mesentery fat tissue, respectively (Supplementary Tables S1–S3). Volcano plots depicting the DEGs are presented in Figure 2. A total of 1,421 (891 upregulated), 5,319 (2,406 upregulated), 875 (489 upregulated), and 1,469 DEGs (816 upregulated) were identified in liver tissue, subcutaneous fat tissue, abdominal fat tissue, and intestinal-mesentery fat tissue, respectively. DEGs involved in lipid metabolism, cell cycle, amino acid metabolism, and anti-inflammation between the control group and the overfeeding group are shown in Supplementary Tables S4–S7. In liver tissue, the gene expression levels of genes involved in fatty acid synthesis were upregulated [stearoyl-CoA desaturase (*SCD*) and elongase of very-long-chain fatty acid family member 6 (*ELOVL6*)], and the gene expression level of lipoprotein lipase (*LPL*) was significantly downregulated. The gene expression level of the cyclin-dependent kinase 1 (*CDK1*) gene involved in the cell cycle was significantly upregulated. In subcutaneous fat tissue, the gene expression level of the *ELOVL1* gene involved in fatty acid synthesis was upregulated, and the gene expression levels of microsomal triglyceride transfer protein (*MTP*) and apolipoprotein B (*APOB*) involved in fatty acid transportation and *β*-oxidation were significantly downregulated. The expression levels of genes involved in complement and coagulation cascade pathways (*C3*, *C4*, *C5*, and *C6*) were significantly downregulated. In abdomen fat tissue, the gene expression level of *LPL* was significantly upregulated. In intestine-mesentery fat tissue, the expression levels of genes involved in glucolipid [*LPL*, carnitine palmitoyltransferase 1 (*CPT1*), and *ELOVL5*] were significantly upregulated. In liver tissue (Figure 3A), the KEGG pathways were mainly enriched in glucolipid metabolism, cell cycle, amino acid metabolism, and inflammation response pathway. In subcutaneous fat tissue (Figure 3B), the KEGG pathway involved in lipid metabolism was mainly enriched in the insulin signaling pathway, phosphatidylinositol signaling system, glycerophospholipid metabolism, and inositol phosphate metabolism. In abdominal adipose tissue (Figure 3C), the significantly enriched KEGG pathway was enriched in glycolysis/gluconeogenesis, ether lipid metabolism, sphingolipid metabolism, glycerophospholipid metabolism, arachidonic acid metabolism, and glycerolipid metabolism. In intestinal-mesentery fat tissue (Figure 3D), the top five KEGG pathways of enrichment were enriched in the phagosome, ECM-receptor interaction, steroid hormone biosynthesis, focal adhesion, and biotin metabolism. GO enrichment analysis was performed to explore the functional group enrichment of the DEGs between the control group and the overfeeding (Supplementary Figures S1–S4). In liver tissue, the biological process was mainly enriched in lipid metabolism. In subcutaneous fat tissue, DEGs were enriched in the biological process involved in the regulation of cell migration, cell motility, and locomotion. In abdominal adipose tissue, the top 10 significantly enriched functional groups were mainly enriched in the regulation of phosphorylation. In intestinal-mesentery fat tissue, the functional groups in the biological process were mainly enriched in the inflammatory response and immune system process.

### Lipidome analysis provides insight into goose fatty liver formation

**Figure 4:**
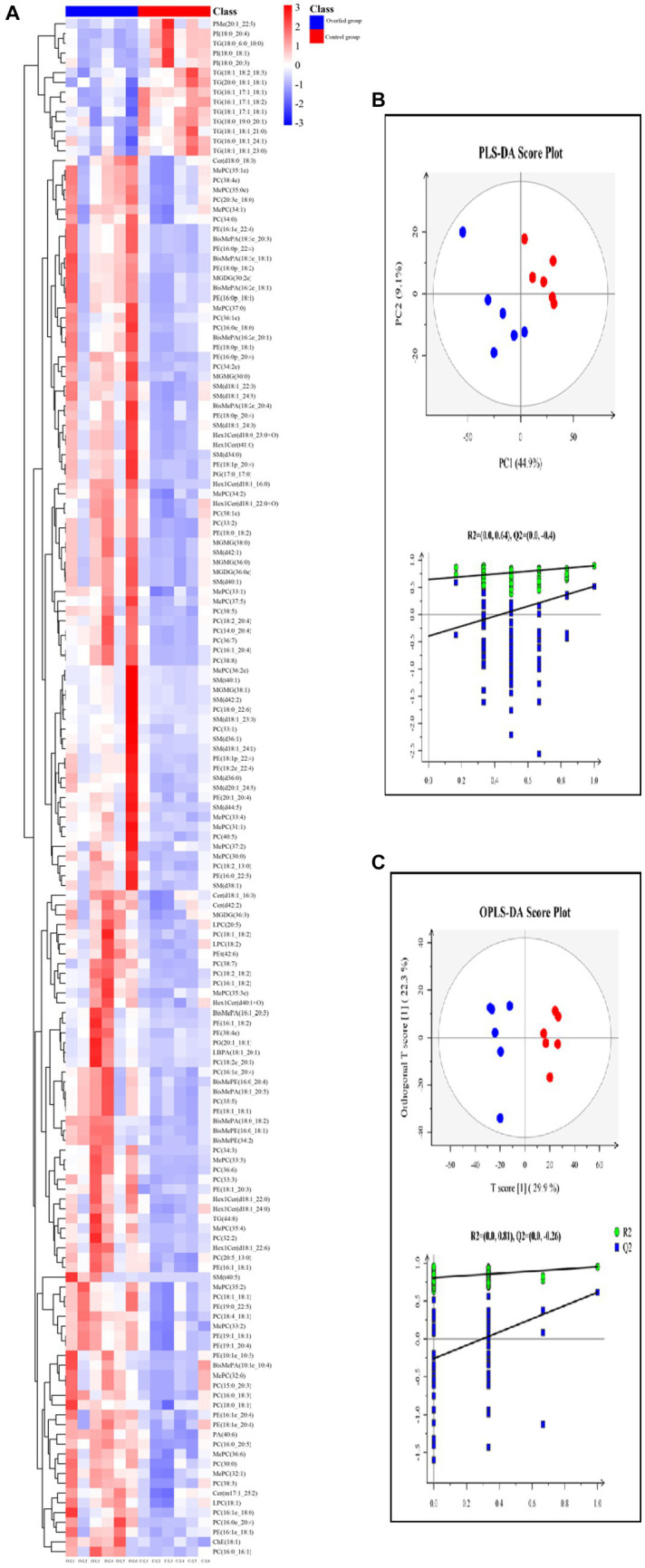
Lipidome analysis of liver (control group vs. overfeeding group) (*n* = 6). **(A)** Hierarchical clustering analysis for the significantly different lipids. **(B)** PLS-DA score plots and corresponding validation plots. **(C)** OPLS-DA score plots and corresponding validation plots. O, overfeeding group; C, control group. The red plot represents the overfeeding group, and the blue plot represents the control group.

In order to better understand the classification and higher level of group separation, the PLS-DA model and OPLS-DA model were used to clarify the different lipidomic patterns. Clear separation and discrimination were found in the PLS-DA and OPLS-DA score plots for comparison. In the PLS-DA model, R2Y and Q2 intercept values were 0.64 and −0.4. The low values of the Q2 intercept represent that the robustness of the model presents a low risk of overfitting and reliability. All Q2 values than 0 in our tests, thereby indicating that the PLS-DA model can identify the difference between groups and be utilized in downstream analysis (Figure 4B). Further permutation tests were performed to validate the OPLS-DA model (R2Y = 0.81, Q2 = −0.26). In the OPLS-DA score plot, the comparison results of the two groups were more obviously separated (Figure 4C).

One hundred and fifty-seven different lipids were yielded. The different lipids are shown in Supplementary Figures S5–S18, which were involved in TG, DG, MGMG, PI, PE, PG, PA, PC, LPC, SM, LPC, Cer, and Hex1Cer. In PC and PE, the different lipids increased after overfeeding (*p* < 0.05), and there was no significant difference between the control group and the corn flour overfeeding group in the PC/PE ratio (2.24 ± 0.15 vs. 2.18 ± 0.34) (*p* > 0.05). The levels of LPC, LPC (18:1), LPC (22:5), and LPC (18:2) increased after overfeeding (*p* < 0.05). The different lipids involved in SM, Cer, and Hex1Cer elevated after overfeeding (*p* < 0.05). The levels of MGDG (32:2e), MGDG (36:0e), and MGDG (36:3) increased, and the levels of MGMG (30:0), MGMG (36:0), MGMG (38:1), and MGMG (38:0) increased after overfeeding (*p* < 0.05). Subsequently, the distinct characteristics of the significantly different lipids were displayed in the hierarchical clustering heat map based on the relative abundance of the identified lipidome (Figure 4A). The *Z*-score plot and the hierarchical clustering heat map presented similar differences in the distribution of lipid content (Supplementary Figure S19).

### Liver-free amino acid change response to overfeeding

**Figure 5:**
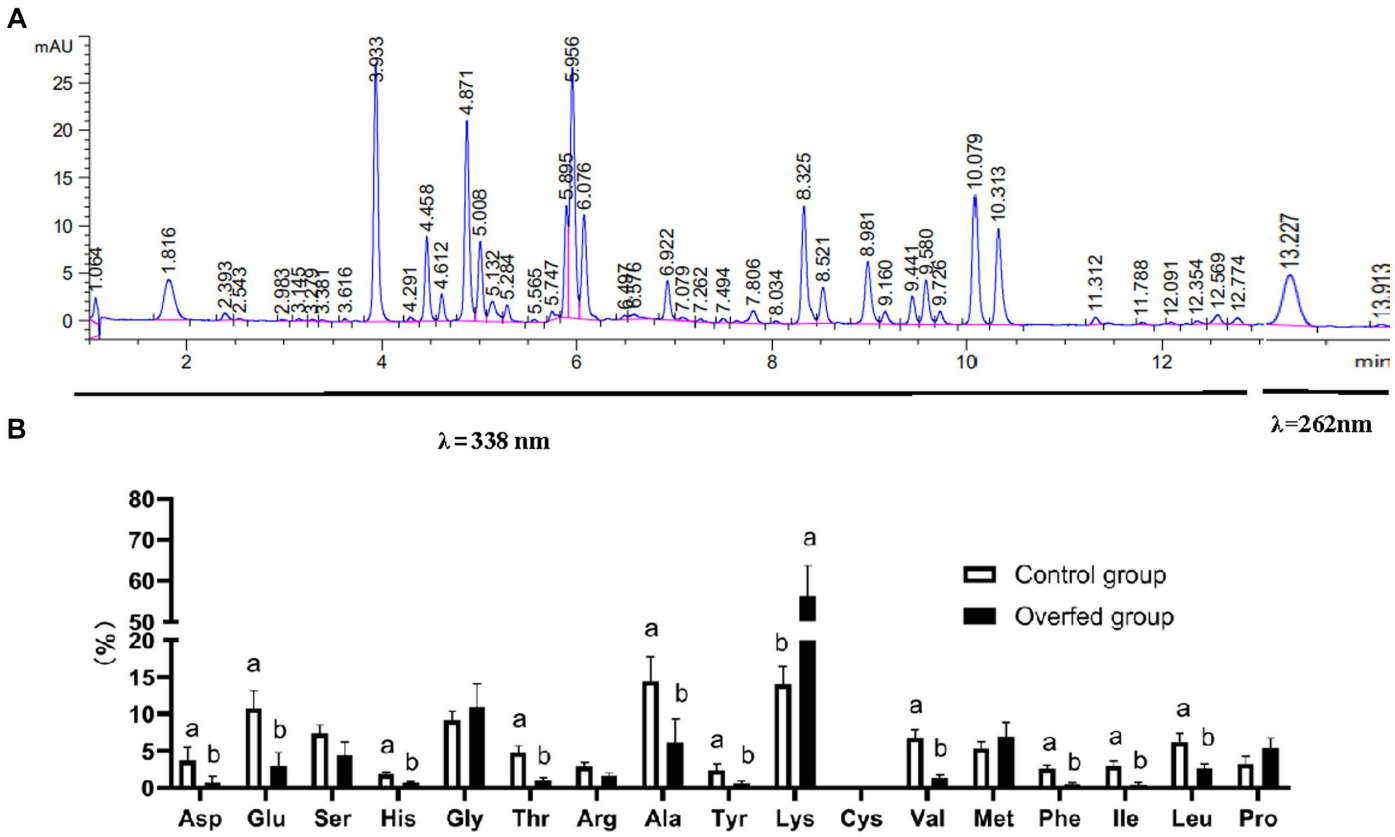
Amino acid metabolome analysis (*n* = 10). **(A)** Amino acid HPLC digital fingerprint. **(B)** Amino acid level difference comparison (control group vs. overfeeding group). 1.064 min, aspartic acid; 1.816 min, glutamic acid; 3.933, serine; 4.612 min, histidine; 4.871 min, glycine: 5.008 min, threonine; 5.895 min, arginine; 5.956 min, alanine; 6.922 min, tyrosine; 8.325 min, lysine; 8.521 min, cystine; 8.981 min, valine; 9.160 min, methionine; 9.441 min, phenylalanine; 9.580 min, isoleucine; 9.358 min, leucine; 13.227 min, proline.

Reversed-phase HPLC by using ortho-phthalaldehyde (OPA) and 9-fluorenyl methyl chloroformate (FMOC) as online derivatization reagent was performed for amino acid metabolome determination. The free amino acid chromatogram of the liver is shown in Figure 5A. In *λ* = 338 nm, these peaks represented primary amino acids (16 amino acids) which were derivatized with OPA. In *λ* = 262 nm, the peak represented a secondary amino acid (only proline) which was derivatized with FMOC. The relative contents were calculated with the area normalization method. The level of Lys was elevated after overfeeding, and the levels of Asp, Glu, His, Thr, Val, Phe, Ile, and Leu were decreased (Figure 5B).

### Integrative analysis of transcriptome, lipidome, and amino acids provides insights into goose fatty liver formation

**Figure 6:**
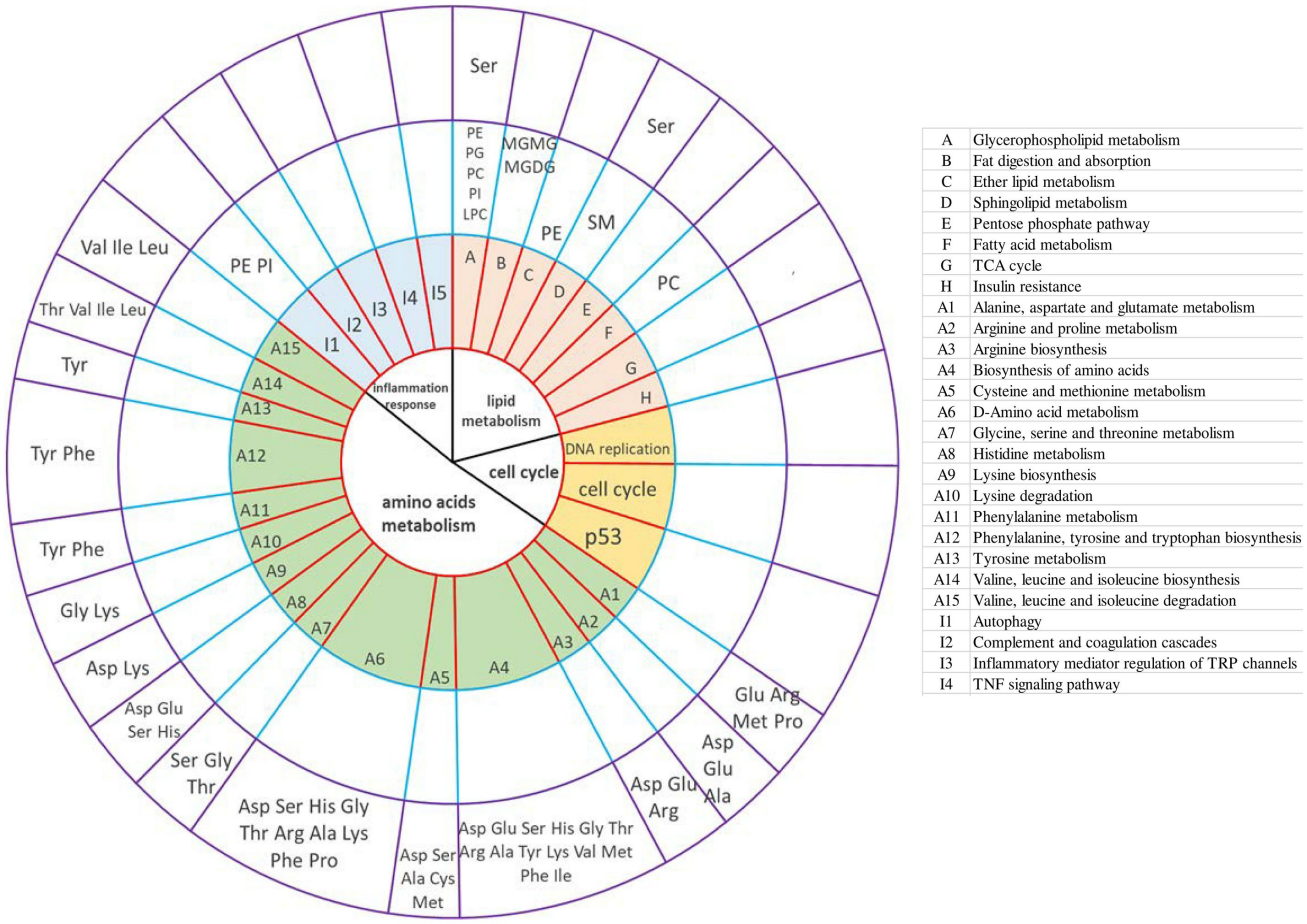
Integrative analysis of the transcriptome, lipidome, and amino acid metabolome provides insight into goose fatty liver formation. The circles from the center outward represented the first-tier pathways (the first circle) and second-tier pathways; KEGG pathways (the second circle) involved in DEGs (control group vs. overfeeding group); different lipids (lipidome) shared the same pathways with the DEGs from liver transcriptome analysis (the third circle); amino acids (amino metabolome) shared the same pathways with the DEGs from liver transcriptome analysis (the fourth circle), respectively. The table on the right illustrates the KEGG pathways (the second circle) involved in DEGs (control group vs. overfeeding group).

The correlation analysis between transcriptome and lipidome is shown in Supplementary Figure S20. The correlation analysis between transcriptome and amino acids showed that lysine was significantly positively correlated with fatty acid-binding proteins (*FABP1*) (glucolipid metabolism) and *CDK1* (cell cycle) (Supplementary Figure S21). The correlation analysis between lipidome and amino acids is shown in Supplementary Figures S22–S26. Lysine and proline were positively associated with the BisMePA, BisMePE, Cer, and Hex1Cer. LPC, MGMG, MGDG, and MePC had a positive correlation with Lys and Pro. MGMG, MGDG, and MePC had a negative correlation with Asp and Glu. PC was positively correlated with Gly, Met, Lys, and Pro and negatively correlated with Asp, Glu, Ala, Val, His, Phe, Arg, Thr, Leu, Ile, and Ser. PE was positively correlated with Lys and Pro and negatively correlated with Asp, Glu, Ala, Val, His, Phe, Arg, Thr, Leu, Ile, and Ser. PI and PMe were negatively correlated with Lys and Pro and positively correlated with Asp, Glu, Ala, Val, His, Phe, Arg, Thr, Leu, Ile, and Ser. SM was negatively correlated with Asp, Glu, Ala, Val, His, Phe, Arg, Thr, Leu, Ile, and Ser and positively correlated with Gly Lys and Pro. TG, except for (TG 44:8), was positively correlated with Asp, Glu, Ala, Val, His, Phe, Arg, Thr, Leu, Ile, and Ser and negatively correlated with Gly Lys and Pro. Integrative analysis showed the interaction between transcriptome, lipidome, and amino acid metabolome (Figure 6). Amnio acids mainly mediate amino acid metabolism, such as D-amino acid metabolism, biosynthesis of amino acids, such as valine, leucine, and isoleucine, and valine, leucine, and isoleucine degradation. In addition, serine-mediated lipid metabolism. Lipids mainly mediate these lipid metabolism pathways: glycerophospholipid metabolism, sphingolipid metabolism, and ether lipid metabolism. PC also mediates fatty acid metabolism. The interrelation between lipidome and lipidome was presented as a chordal graph (Supplementary Figure S27). Notably, PE and PI mediated the autophagy pathway.

## Discussion

After overfeeding, the goose received large amounts of carbohydrates, as a result of which the blood glucose elevated significantly, and then, glucose was converted to lipid. When the speed of lipid synthesis far exceeded the transport speed of lipids, and the capacity of fatty acid synthesis far exceeded *β*-oxidation, thus leading to lipid accumulation in goose liver. Transcriptome analysis showed that the gene expression levels of key enzymes involved in hepatocyte fatty acid synthesis (*ELOVL6* and *SCD*) were significantly elevated, while the gene expression level of the liver lipoprotein lipase (*LPL*) was significantly downregulated. These results are in line with previous studies reported by Lu et al. (12). *ELOVL6* is the rate-limiting enzyme of long-chain fatty acid elongation reaction. Hepatic *ELOVL6* expression level was highly upregulated in ob/ob mice deficient in leptin (22). A recent study suggested that the large amount of fat stored in goose liver resulted from an imbalance between the storage and secretion of *de novo* synthesized endogenous lipids and an absence of leptin gene homologs due to positive selection (12). This long-chain fatty acid elongation enzyme is a new member of lipid synthetase regulated by *SREBP1*, which plays an important role in the *de novo* synthesis of long-chain saturated fatty acids and monounsaturated fatty acids and is associated with *SCD1* and fatty acid synthetase gene (*FAS*) (23). Continuous activation of *ELOVL6* caused the nuclear sterol-regulatory element binding protein (*SREBP*) overexpression in transgenic mouse livers (24, 25). *SREBP* has three isoforms, *SREBP1a*, *SREBP1c*, and *SREBP2*, which play different roles in lipid synthesis. *SREBP2* regulates cholesterol synthesis in the lytic system, while *SREBP1c* regulates adipogenesis mainly by changing *SREBP1c* mRNA levels (26). Our previous research showed that the level of polyunsaturated fatty acids significantly increased in the process of goose fatty liver formation (17). This may be the reason why *SREBP2* was downregulated as shown in liver DEGs. Previous studies had confirmed polyunsaturated fatty acids inhibited the abundance of *SREBP2*, thereby reducing the expression of low lipid synthetic genes *in vitro* (27). Carbohydrate response element-binding protein (*ChREBP*) is a key transcription factor regulating glucolipid metabolism. Previous studies had confirmed that *ChREBP* promoted lipid synthesis via mediating its target gene expression [acetyl CoA carboxylase (*ACC*) and fatty acid synthase (*FAS*)] and then led to NAFLD (22). *ChREBP* usually plays a transcriptional regulatory role in the form of a heterodimer with Max-like protein X (*MLX*) (28). Our DEGs showed that *MLX* was upregulated after overfeeding, which was consistent with the glucolipid metabolism change in goose fatty liver formation. The process of goose fatty liver formation is closely associated with endoplasmic reticulum stress (ERS), IR, and hepatocyte growth and proliferation. Liver transcriptome KEGG analysis showed that the DEGs involved in the cell cycle, TP53, and DNA replication pathways were enriched, which suggested that cell proliferation occurred in the goose liver. Cyclin-dependent kinase (*CDKs*) is the core of the entire cell cycle regulatory protein. *CDKs* can only be activated to perform their functions after combining with the corresponding cell cycle regulatory protein to form a CDK-cyclin complex (29). In this experiment, the gene expression of *CDK1* was significantly upregulated in the overfeeding group, indicating that overfeeding promoted the mitosis of hepatocytes and induced the proliferation and meristem of hepatocytes.

The interaction between peripheral adipose tissue and the liver plays a role in the development of NAFLD. Adipose tissue can release molecules (adipokines) that regulate lipid metabolism, interact with insulin sensitivity, and may contribute to inducing fibrogenesis in the liver (18); in addition, adipose tissue, especially visceral adipose tissue, comprises multiple cell populations that produce adipokines and insulin-like growth factor, plus macrophages and other immune cells that stimulate the development of lipotoxic liver disease (19). A study on pigs reported that high-fructose feeding upregulated hepatic *de novo* lipogenesis enzymes, and pigs utilize adipose tissue as the main *de novo* lipogenesis organ based on substantially higher *ACACA* and *FAS* protein levels (per total protein) compared to hepatic protein levels (20). Therefore, the fat deposition in peripheral adipose tissue is a protective mechanism that protects the liver from the harm caused by severe hepatic steatosis. In this current study, abdominal fat tissue transcriptome analysis showed that *LPL* was upregulated. In intestine-mesentery fat tissue, the DEGs involved in glucolipid (*LPL*, *CPT1*, and *ELOVL5*) were significantly upregulated. The lipids produced in the liver are mostly transported by the very low-density lipoproteins (*VLDL*), and the lipids in the diet are primarily transported by chylomicrons (12). A majority of the chylomicrons and lipids transported by *VLDL* are deposited in peripheral adipose tissue and muscle after being hydrolyzed by *LPL* present in the blood. Nevertheless, if the lipid transported by lipoprotein is not hydrolyzed by *LPL*, it will be transported to the liver for deposition as mediated by special lipoprotein receptors in the liver, thus facilitating the formation of fatty liver. *LPL* was significantly downregulated in the liver. These DEGs suggested that fewer liver lipids were ex-transported, and fewer lipids were accumulated in peripheral adipose tissue, and more lipids accumulated in overfed goose liver. Therefore, the interaction between peripheral adipose tissue and the liver cooperatively promoted lipid accumulation in the goose liver during overfeeding.

Glycerophospholipids, sterols, and sphingolipids are the major skeleton of membranes and contribute to the cellular physiological process as well as signal transduction. Moreover, after overfeeding, we observed differences in goose liver lipids (TG, SM, PI, PE, PC, MGMG, DG, LPC, CL, Cer, BisMePA, BisMePE, deMePE, MePC, and Hex1Cer). The PC/PE molar ratio is a determinant of cell membrane integrity and a predictor of NAFLD. Previous studies have demonstrated that a decrease in the PC/PE ratio in phosphatidylethanolamine N-methyltransferase-knockout mice (Pemt−/−) led to a loss of membrane integrity, followed by hepatic damage (such as ballooning) and progression to NASH (30). In this study, there was no significant difference between the control group and the overfeeding group in the PC/PE ratio (2.24 ± 0.15 vs. 2.18 ± 0.34), which suggested that cell membrane integrity was not lost when excessive lipids deposited in the liver and promoted hepatocyte growth and proliferation. Numerous liver diseases have been found to cause changes in plasma LPC levels, making LPC a potential biomarker for liver disease (31, 32). In this study, LPC (18:1), LPC (22:5), and LPC (18:2) were significantly increased. It suggested that the chronic hepatitis occurred in the goose fatty liver, which was consistent with our previous report (17). It is worth noting that when the overfed geese were fed a regular diet within a 20 days period of recovery, and the migratory birds after energy consumption of stored lipids, their liver was restored to the original state (15). The entire process was found to be reversible. In addition, it was discovered to cause neither cirrhosis nor necrosis in the liver, suggesting that waterfowl have a mechanism to protect their livers from the harm caused by severe hepatic steatosis. Autophagy is a common metabolic process in most eukaryotic cells, which can be used as a self-protection mechanism. Autophagy can inhibit inflammation (33, 34). In the current study, the result of multi-omics integrative analysis showed PE mediated the autophagy pathway, and the PE level was significantly elevated.

Amino acids can be converted into lipids and carbohydrates by gluconeogenesis after deamination, which means that amino acids can not only be used as the basic building blocks of proteins but also can be converted into fats and carbohydrates. Meanwhile, amino acid is also an important signal molecule regulating nutrient metabolism. It is of great significance to study the regulation of amino acids on lipid metabolism. L-serine can catalyze the metabolism of fat and fatty acids. Moreover, L-serine can be degraded to pyruvate and participate in the TCA cycle, providing the body with a large amount of energy (35). This is consistent with our integrative analysis. Integrative analysis of the transcriptome, lipidome, and amino acid metabolome showed that serine mediated lipid metabolism (Figure 6). Leu, Ile, and Val are branched-chain amino acids (BCAA). Previous studies have shown that BCAAs were required for insulin release (36, 37). When BCAA levels are elevated in circulation, insulin resistance occurs. In obese patients and obese animal models, the activity of branched-chain ketoacid dehydrogenase (*BCKD*) (mainly muscle and liver) decreased, which led to the elevation of circulating BCAA levels. Elevated circulating BCAAs can lead to insulin resistance by interfering with lipid oxidation in skeletal muscle and activating the mTOR pathway (38, 39). A large number of reports have confirmed that insulin resistance is one of the triggers of inflammation (40). Our results showed that the level of Val, Ile, and Leu decreased in geese liver after overfeeding. Thus, it can be speculated that the decreased BCAA downregulated the insulin resistance in liver tissue, and then restricted the inflammation response, which may be one of the underlying protective mechanisms that protect the liver from the harms caused by severe hepatic steatosis in the process of goose fatty liver formation. However, further investigation is needed to verify the speculation. Interestingly, only lysine level was elevated in these 17 free amino acids. It may be selected as the potential biomarker for the diagnosis and treatment of liver diseases.

## Conclusion

This study integrated transcriptome, lipidome, and amino acid metabolome, which provided not only a comprehensive understanding of goose fatty liver formation from glucolipid and amino acid metabolism but also a detailed insight into goose liver capacity for tolerance to severe hepatic steatosis from metabolic reprogramming. Goose fatty liver has a special hepatic steatosis process where lipid deposition accompanies cell proliferation. Enriched pathways and DEGs involved in lipid metabolism and cell cycle were identified by liver transcriptome. Peripheral adipose tissue also mediated the process of goose fatty liver formation. Lipids and amino acid metabolic reprogramming cooperatively promoted goose fatty liver formation during overfeeding, and the different lipids and free amino acids jointly shaped the liver metabolism landscape.

## Data availability statement

The datasets presented in this study can be found in online repositories. The names of the repository/repositories and accession number(s) can be found below: 10.6084/m9.figshare.21154540.

## Ethics statement

All procedures in the present study were subject to approval by the Institutional Animal Care and Use Committee (IACUC) of Sichuan Agricultural University (Permit No. DKY-B20141401), and carried out in accordance with the approved guidelines. Written informed consent was obtained from the owners for the participation of their animals in this study.

## Author contributions

RW: methodology, investigation, data curation, writing original draft, and formal analysis. CH: conceptualization, methodology, resources, supervision, project administration, funding acquisition, and writing—review and editing. SW and LL: methodology, investigation, and data curation. YT, SH, and HL: investigation. BK: resources. HX: funding acquisition. All authors contributed to the article and approved the submitted version.
