# Supplementary material for: Multi-omics reveals goose fatty liver formation from metabolic reprogramming
Source: Front Vet Sci. 2024 Jan 29;11:1122904. doi: 10.3389/fvets.2024.1122904 (PMC10859500; doi:10.3389/fvets.2024.1122904)
Supplement: Supplementary file 1 [file Data_Sheet_1.docx]

High-performance Liquid Chromatograph (HPLC) condition:

Experimental

Apparatus and reagents

Agilent 1260 Infinity LC equipped with the following Agilent components:

• G1311C 1260 Quat Pump VL

• G1329B Autosampler 1260 ALS

• G1316A 1260 TCC

• G1315 D 1260 DAD

Parameter Value

Chromatograph Column: ZORBAX Eclipse Plus 95Å C18, 4.6 x 100 mm, 3.5 µm HPLC column (959961-902)

Guard cartridge: ZORBAX Eclipse Plus 95Å C18, 5 µm, 4.6 x 12.5 mm (820950-936).

Flow rate: 1.5 mL/min

Column temperature: 40 °C

Mobile phases:

(A) Water

(B) Methyl alcohol

(C) Methanol:acetonitrile:water = 45:45:10 (v:v:v)

(D) 10 mM Dibasic sodium phosphate and 10 mM sodium borate, pH adjusted to 8.2 with 6 M hydrochloric acid.

(Mobile phase A and B were used to wash and activate column. Mobile C and D were used to perform amino acid detection. Mobile phase solutions were filtered through a 0.22-μm filter membrane with extraction filtration).

Gradient program:

0.0 - 0.35 min: 2% mobile phase C;

0.35 -13.4 min: 2% - 57% mobile phase C;

13.4-13.5min: 57% - 100% mobile phase C;

13.5-15.7 min: 100% mobile phase C;

15.7-15.8 min: 100% - 2% mobile phase C;

15.8-18.0min: 2 mobile phase C.

Detector

338 nm, 10 nm bandwidth (primary amino acids)

262 nm, 16 nm bandwidth (secondary amino acids)

Derivative program

The automated online derivatization can easily be achieved using an Agilent autosampler. The primary amino acids were derived from o-phthalaldehyde (OPA), and the secondary amino acids were derived from 9-fluorenylmethyl chloroformate (FMOC).

Derivatization reagent:

Borate saline buffer（0.4M,pH=10.5: 2.48g boric acid + 1.41g sodium hydroxide，dissolved in water and bring to volume in 100ml volumetric flask.

Phthalaldehyde solution（OPA）：50mg/ml (methanol as solvent)

OPA/MPA Solution：0.8ml borate saline buffer - 0.2ml OPA solution -20μl 3-mercaptopropionic acid

Fluorenylmethyl chloroformate solution（FMOC）：2.5mg/ml（acetonitrile as solvent）

Diluent：Mobile phases D

Autosampler procedure

• Draw 5 µL from a borate vial (1).

• Draw 1.0 µL from a sample vial (from Svample Vial).

• Mix 6 µL in wash port, five times.

• Draw 0 µL from a water (2).

• Wait 0.2 minutes, then draw 1 µL of OPA(3).

• Mix 7 µL at wash port, 10 times.

• Draw 0 µL from a water (2).

• Draw 1 µL of FMOC (4).

• Mix 8 µL at wash port, 10 times.

• Draw 0 µL from a water (2).

• Draw 8 µL from a diluent vial (5).

• Mix 16 µL in wash port, eight times.

• Inject the sample.

Note: The step “Draw 0 µL from a water (2)” is equal to wash the injection needle procedure.

(1), (2), (3),(4) and (5) represent position of vial, individually;

(1) = borate; (2) = water; (3) = OPA; (4) = FMOC; (5) = diluent vial

Derivatization reagent and sample were filtered through a 0.22-μm filter membrane.
