# Supplementary material for: Multi-omics reveals goose fatty liver formation from metabolic reprogramming
Source: Front Vet Sci. 2024 Jan 29;11:1122904. doi: 10.3389/fvets.2024.1122904 (PMC10859500; doi:10.3389/fvets.2024.1122904)
Supplement: Supplementary file 2 [file Data_Sheet_2.pdf]

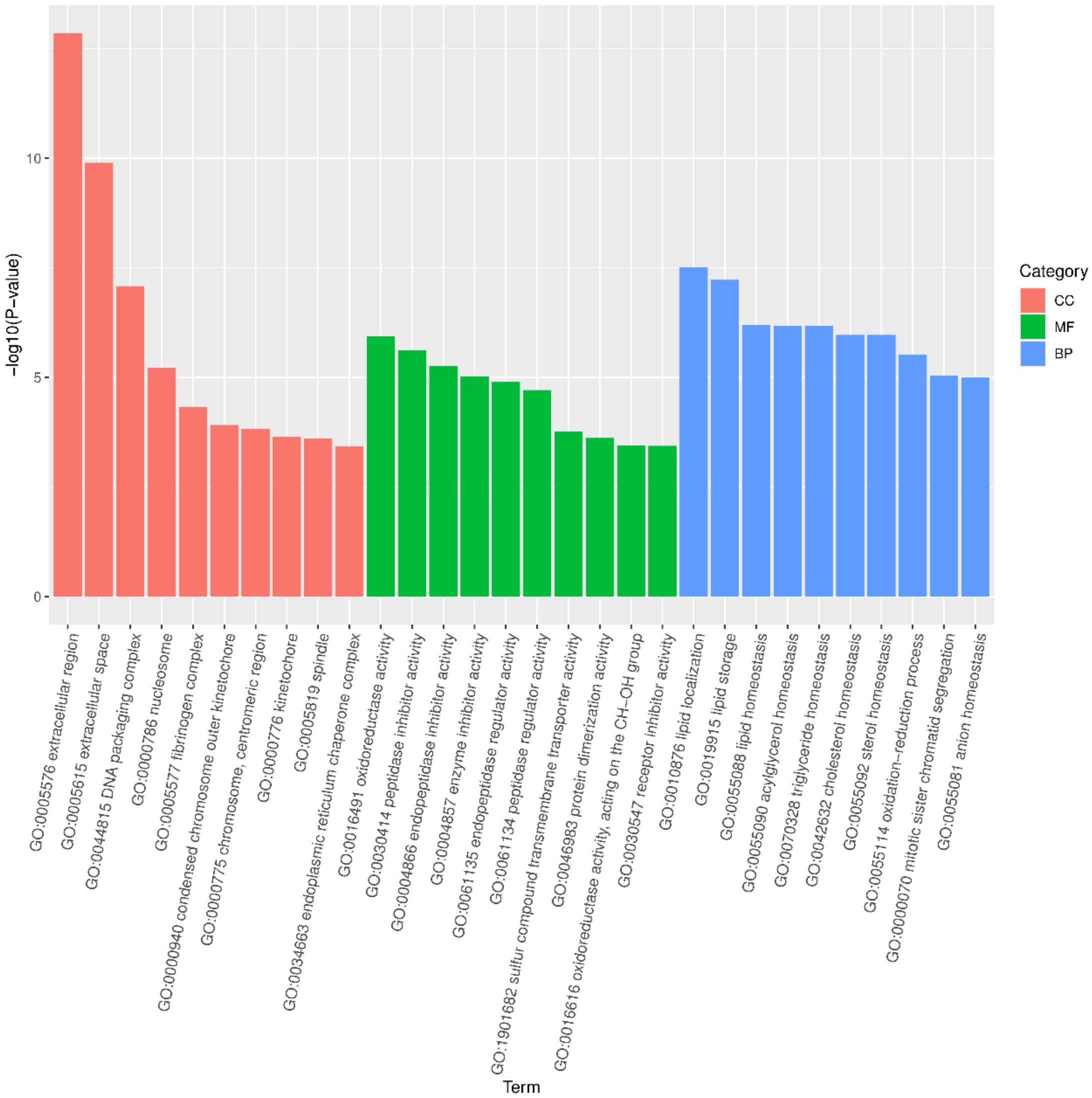

Figure S1 GO analysis of liver tissue

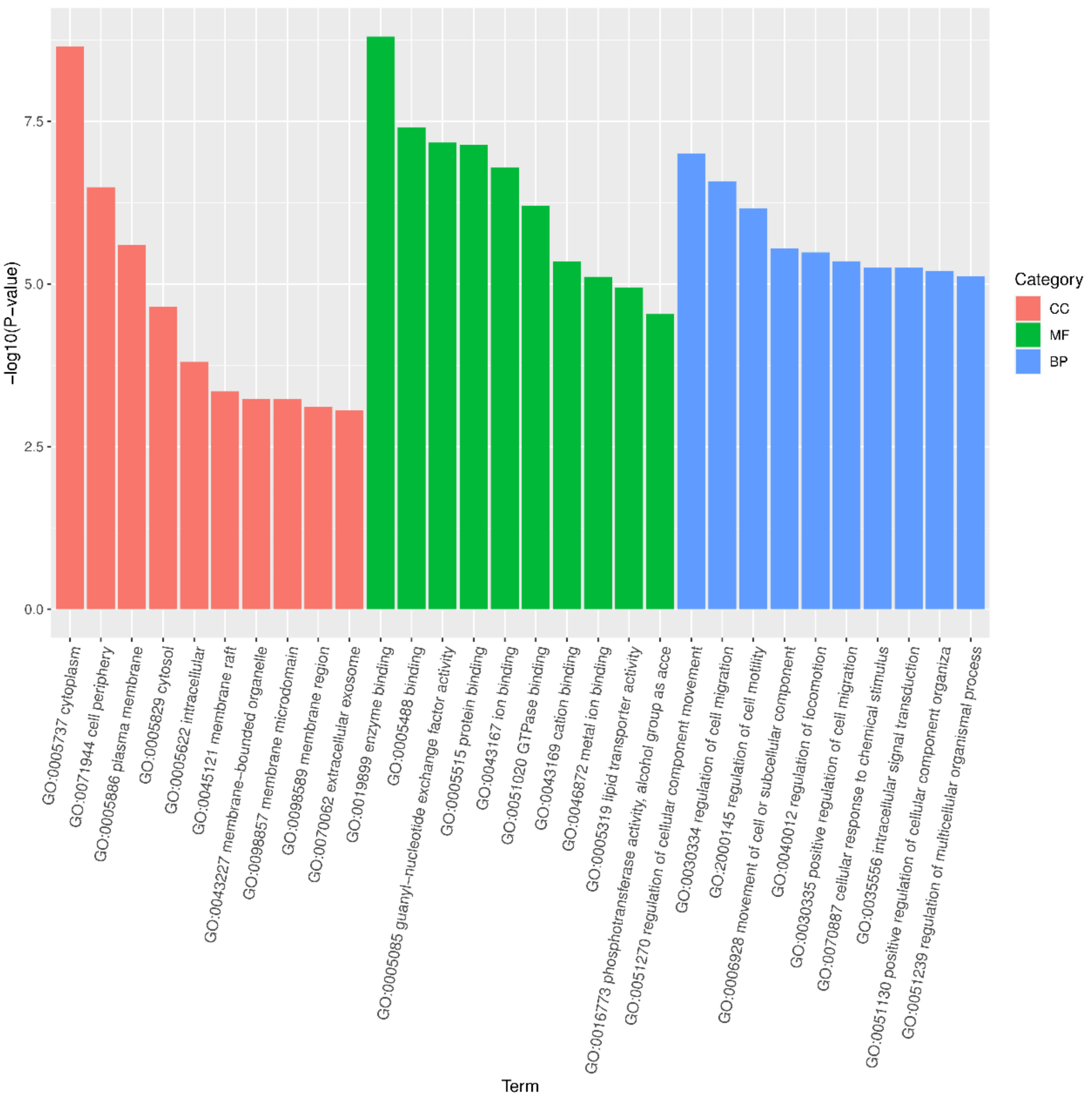

Figure S2 GO analysis of subcutaneous fat tissue

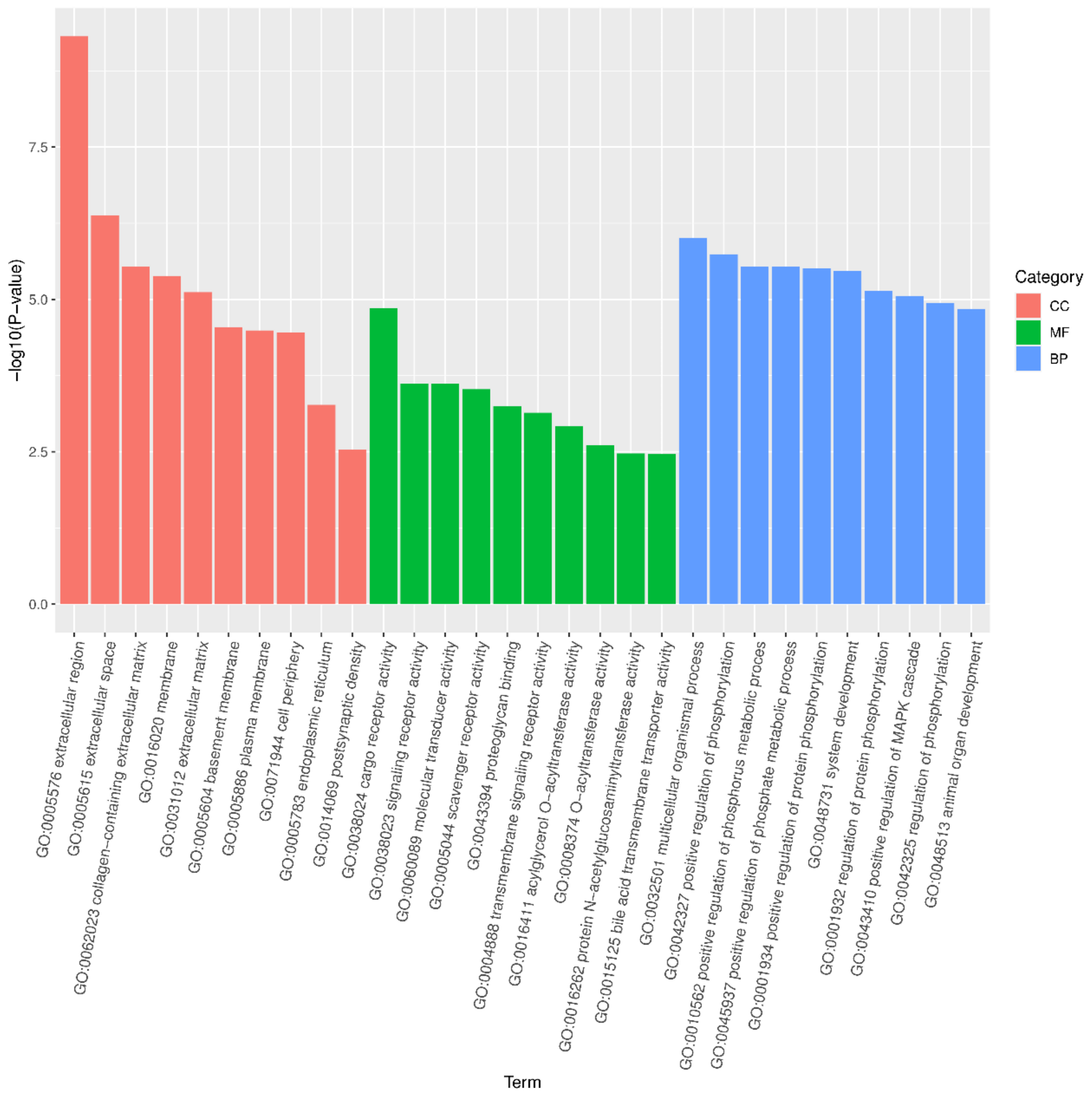

Figure S3 GO analysis of abdomen fat tissue

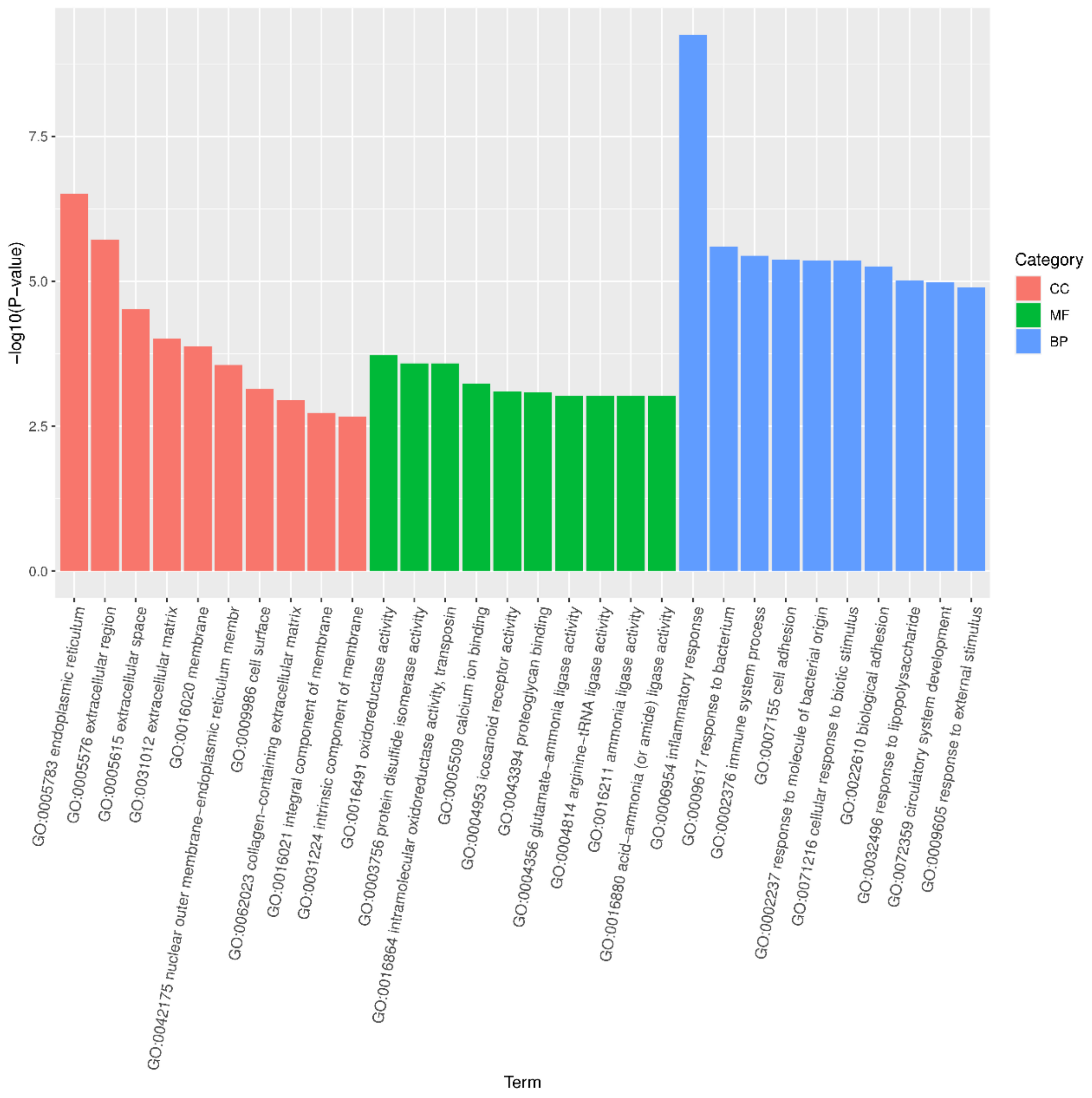

Figure S4 GO analysis of intestine-mesentery tissue

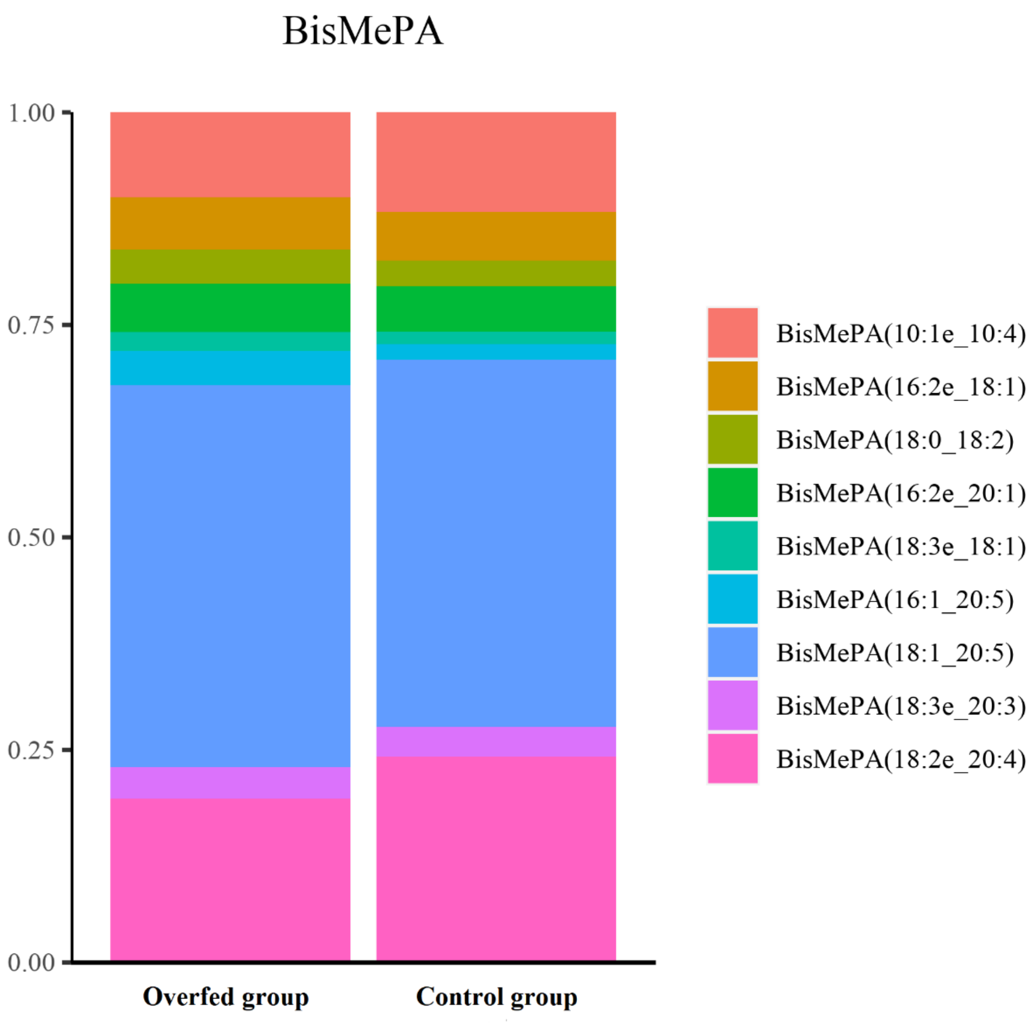

Figure S5 Liver lipidome different lipids- BisMePA

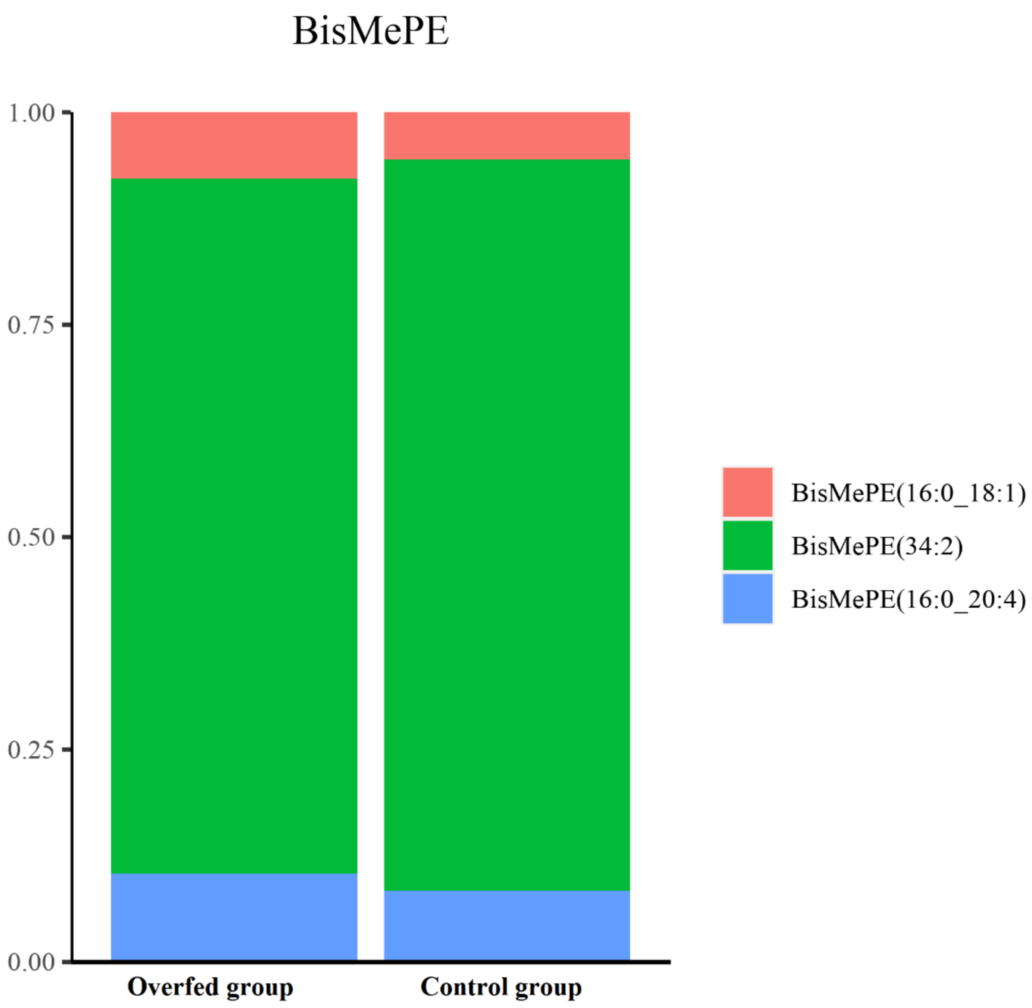

Figure S6 Liver lipidome different lipids- BisMePE

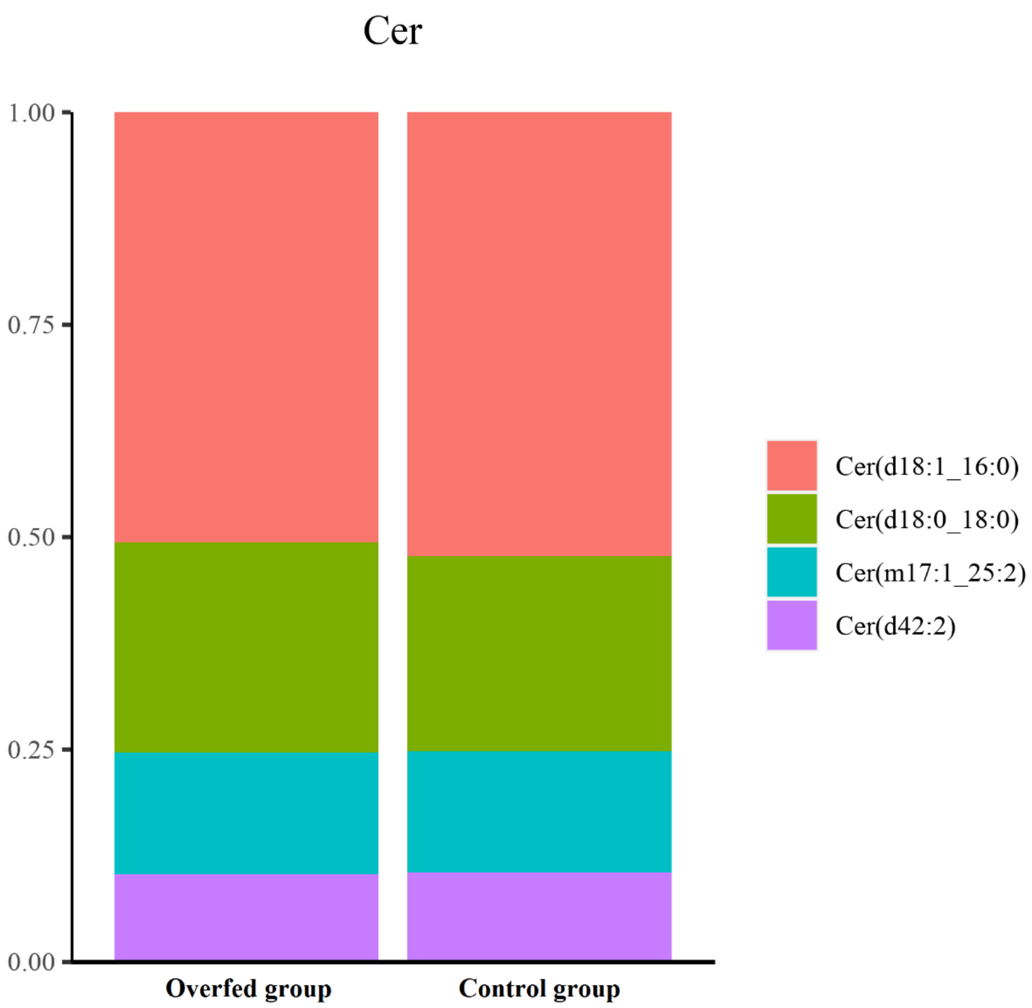

Figure S7 Liver lipidome different lipids-Cer

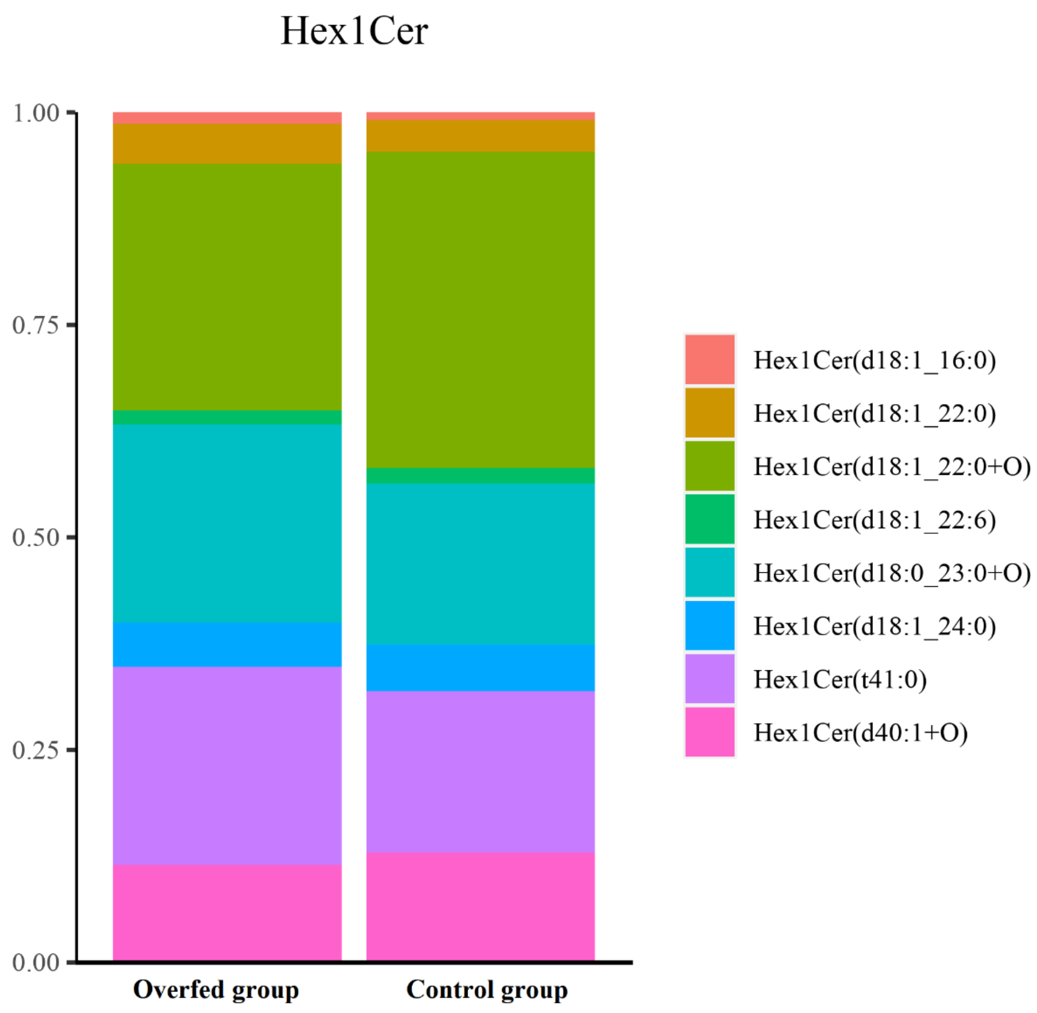

Figure S8 Liver lipidome different lipids- Hex1Cer

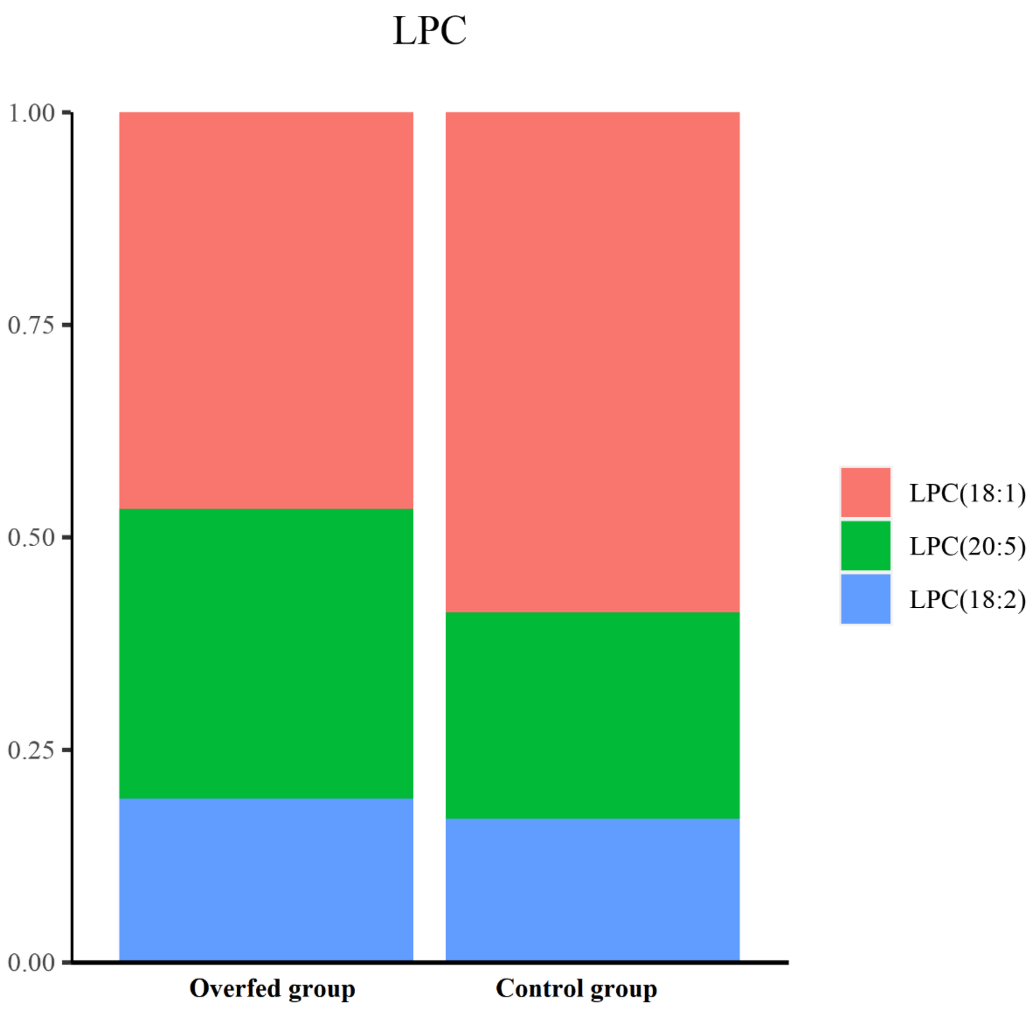

Figure S9 Liver lipidome different lipids-LPC

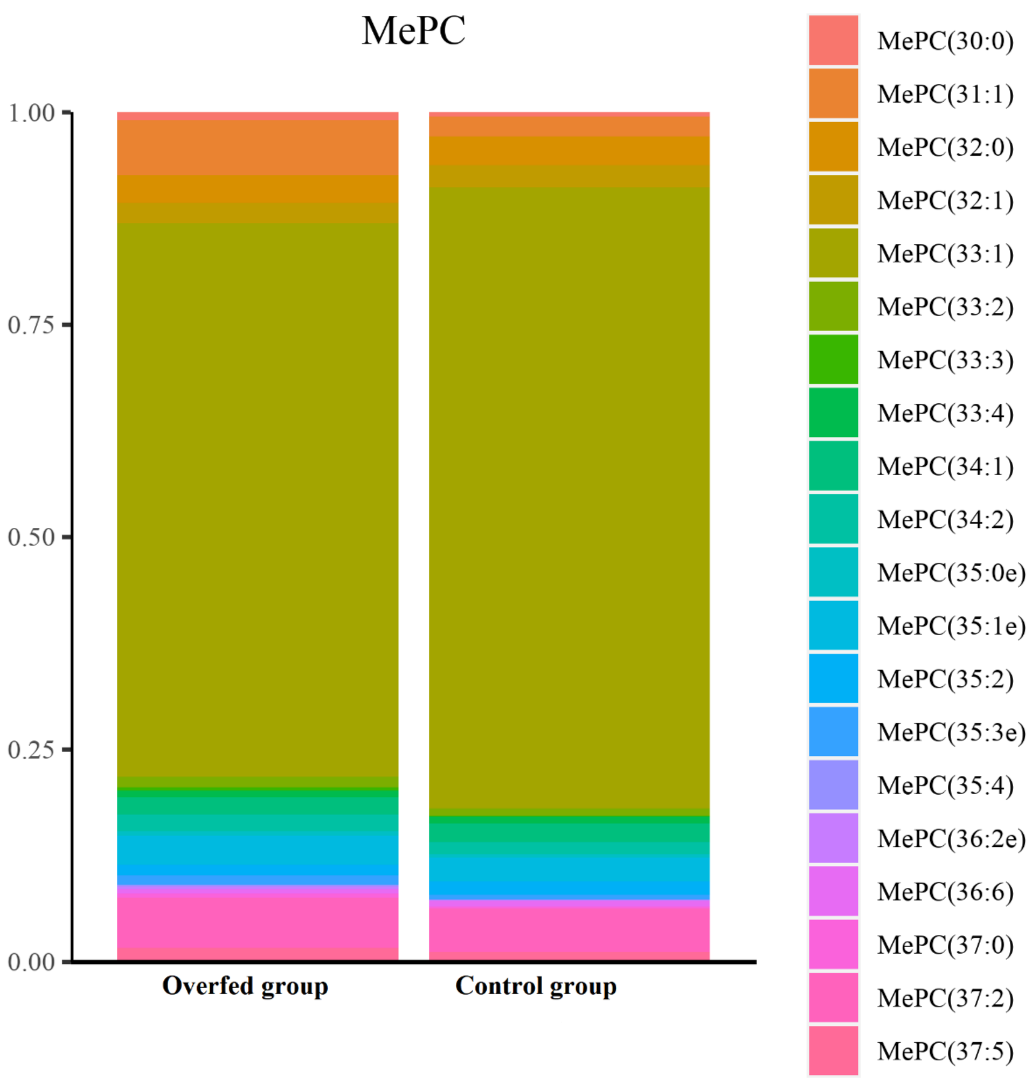

Figure S10 Liver lipidome different lipids-MePC

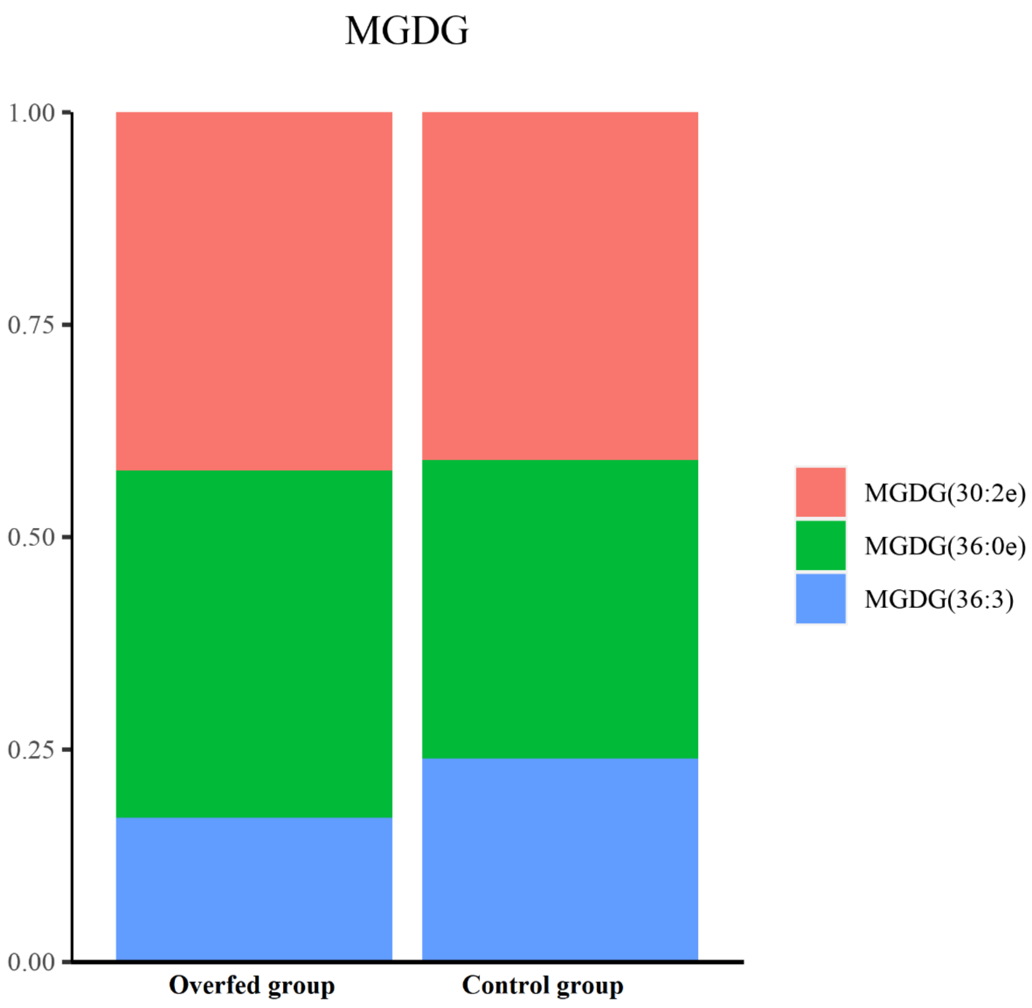

Figure S11 Liver lipidome different lipids-MGDG

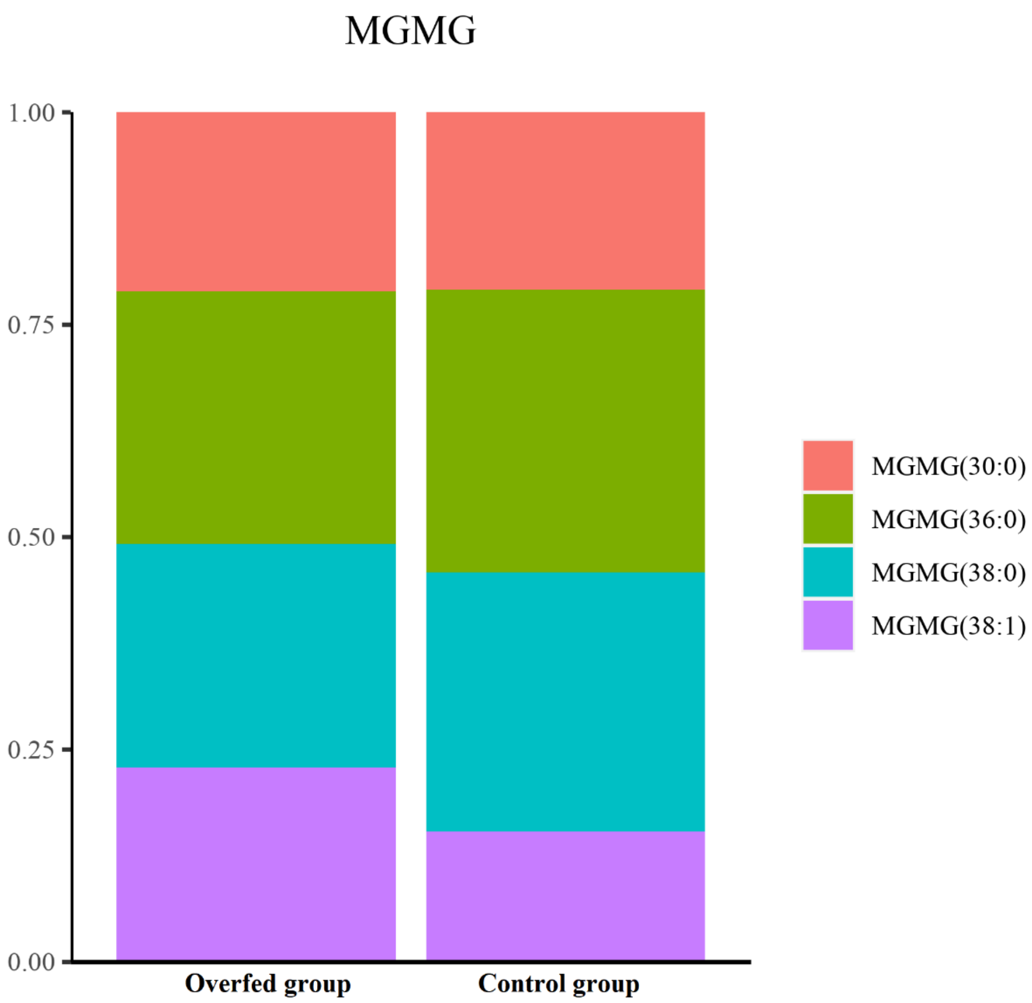

Figure S12 Liver lipidome different lipids-MGMG

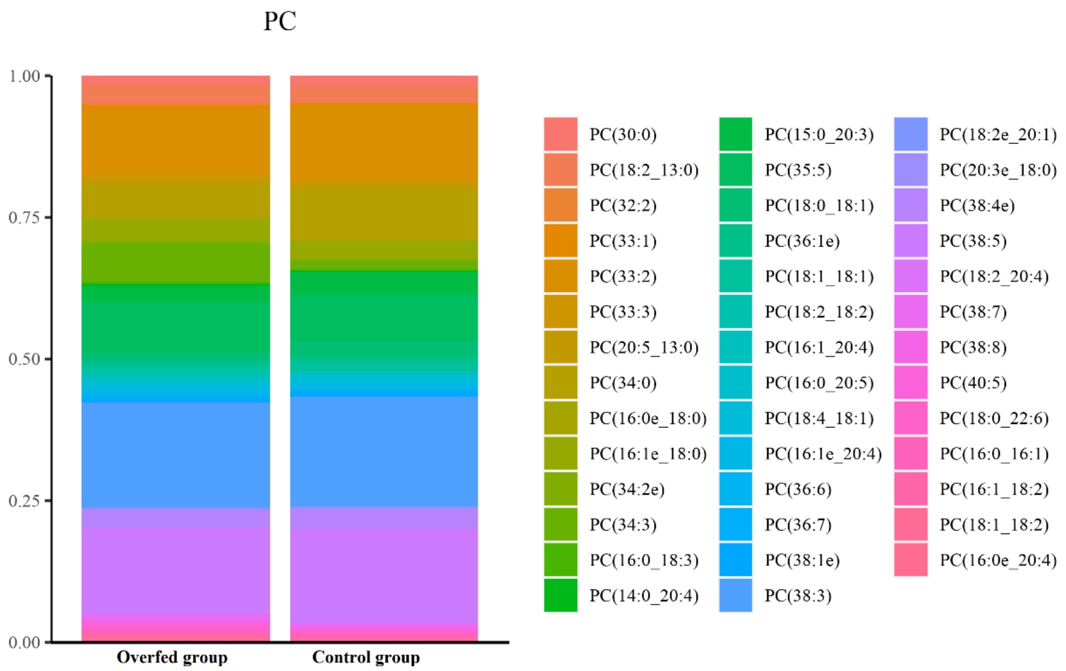

Figure S13 Liver lipidome different lipids-PC

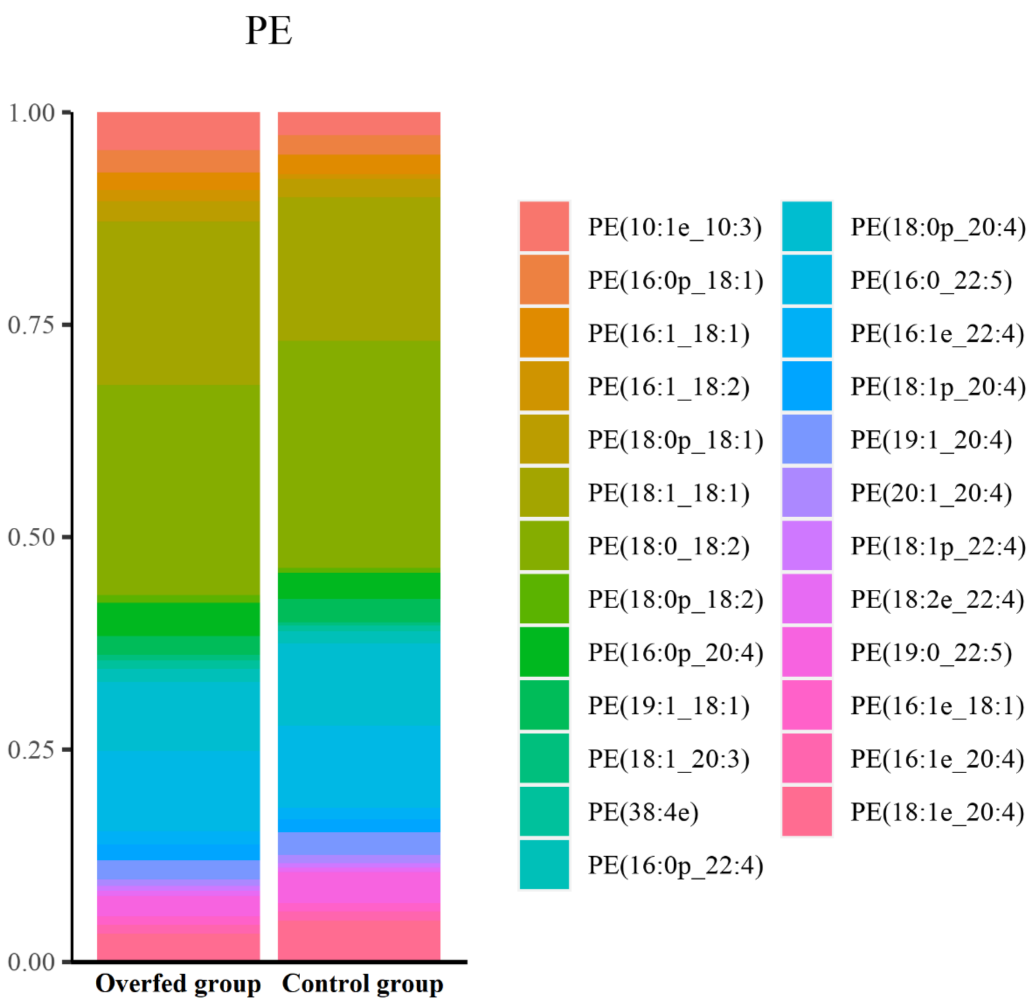

Figure S14 Liver lipidome different lipids-PE

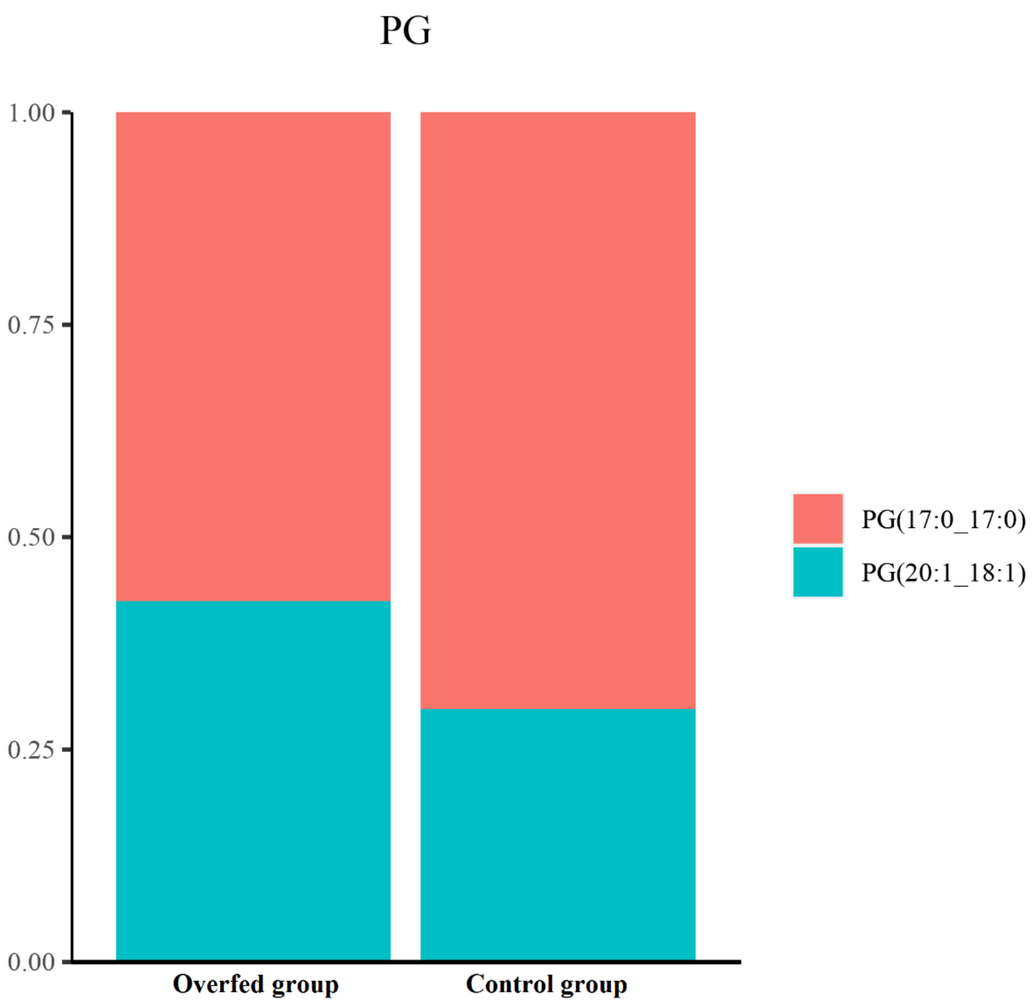

Figure S15 Liver lipidome different lipids-PG

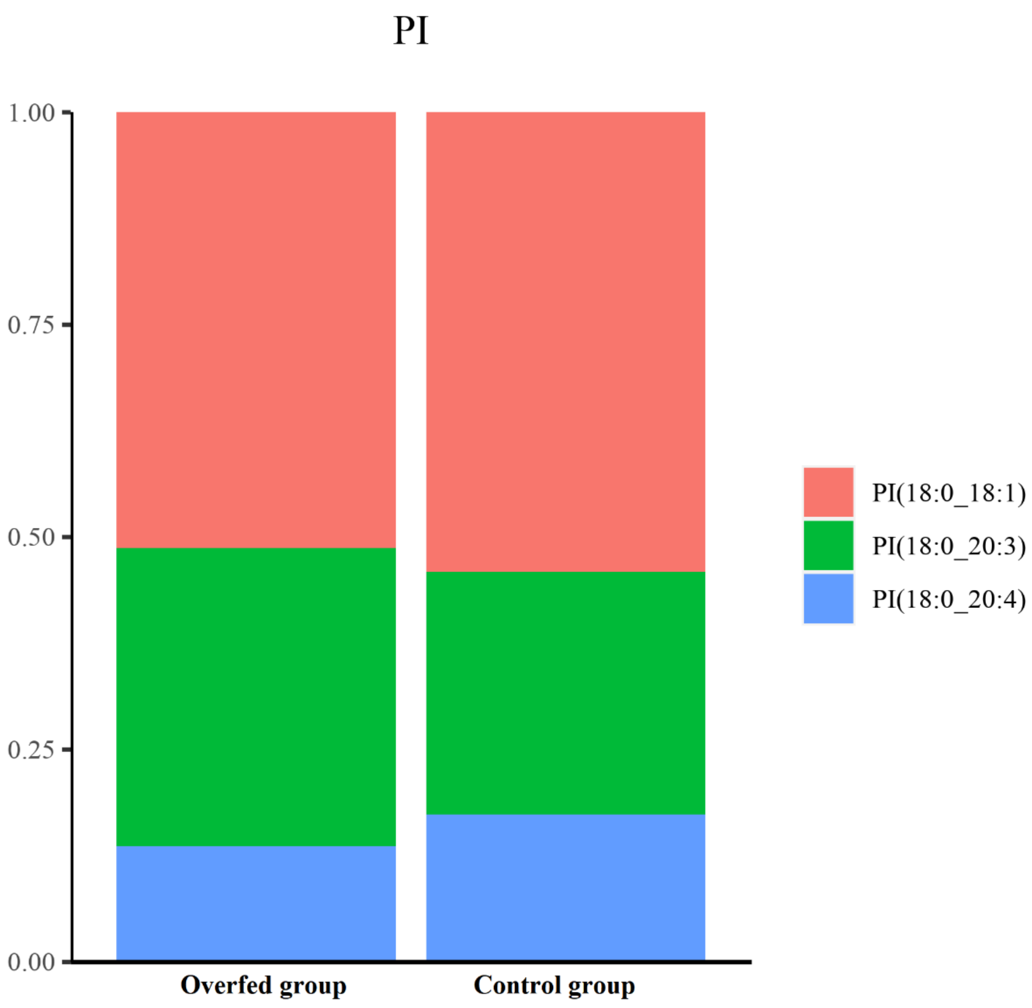

Figure S16 Liver lipidome different lipids-PI

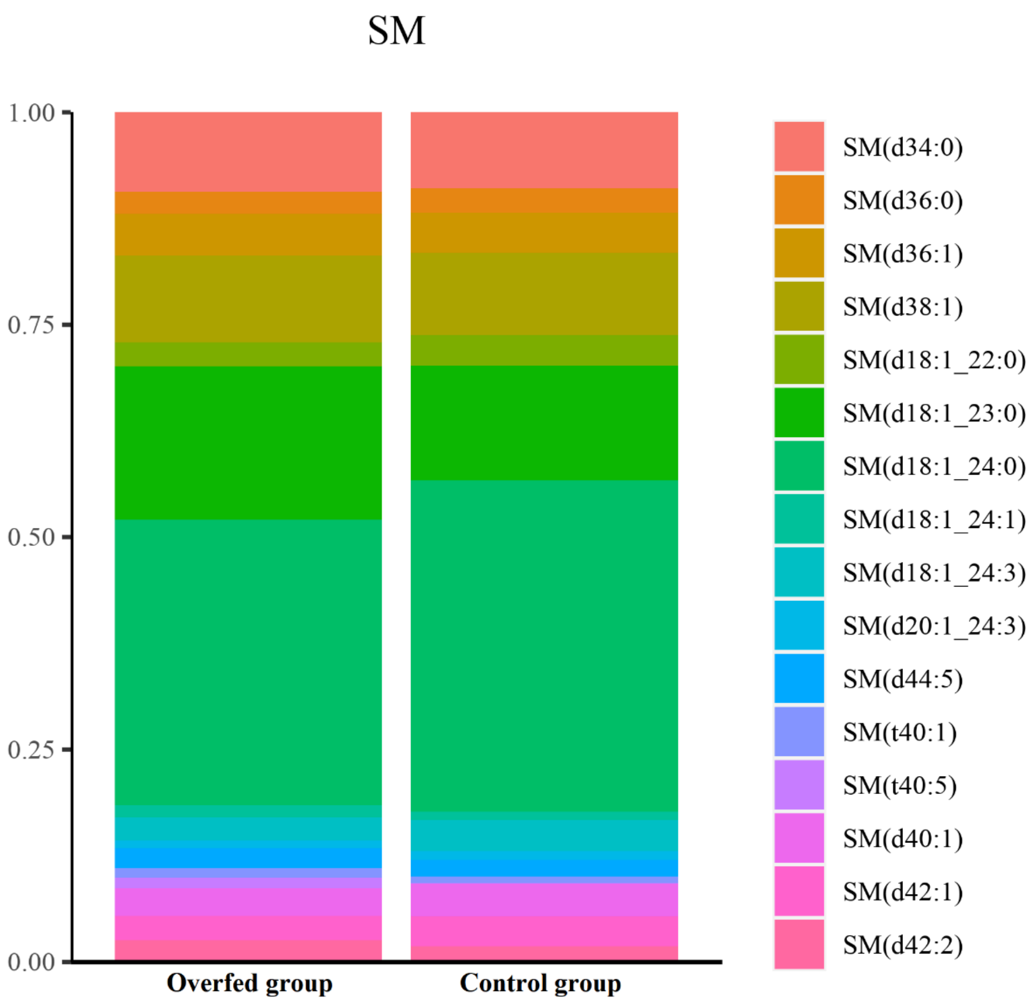

Figure S17 Liver lipidome different lipids-SM

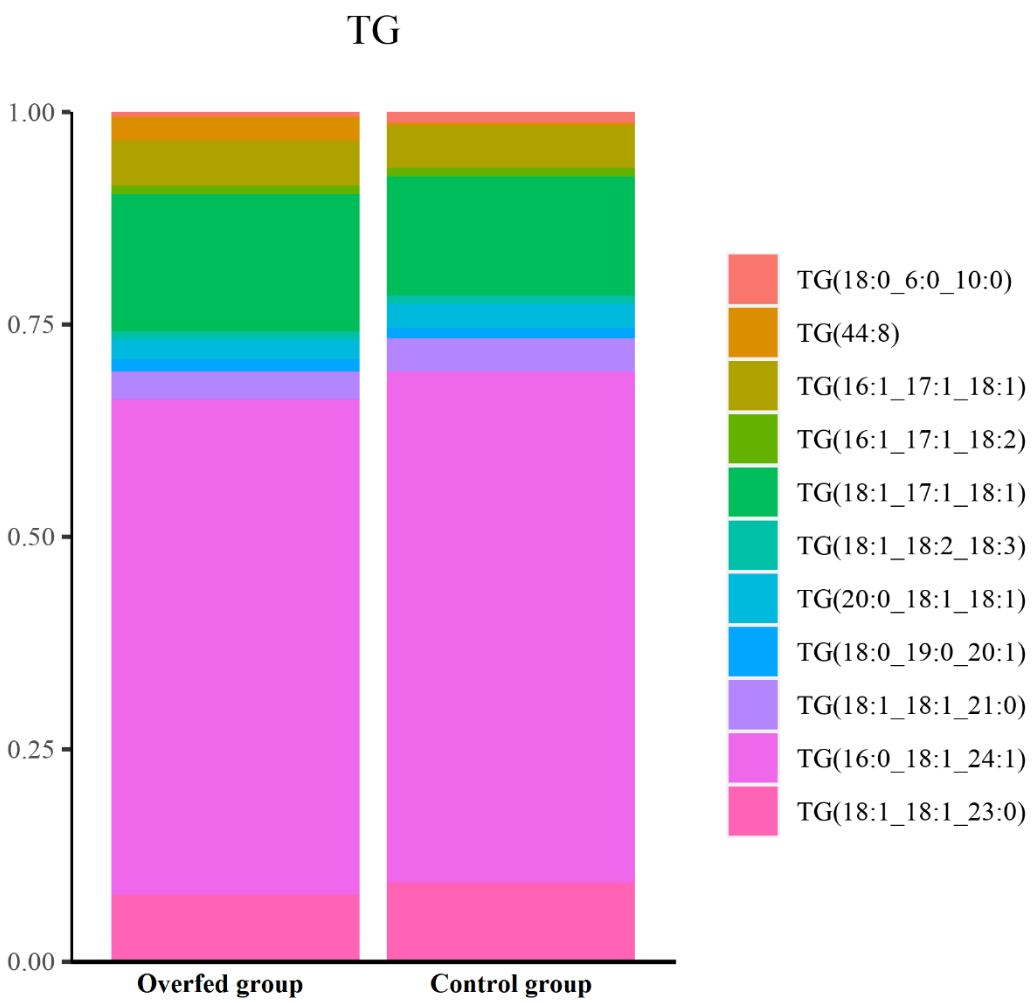

Figure S18 Liver lipidome different lipids-TG

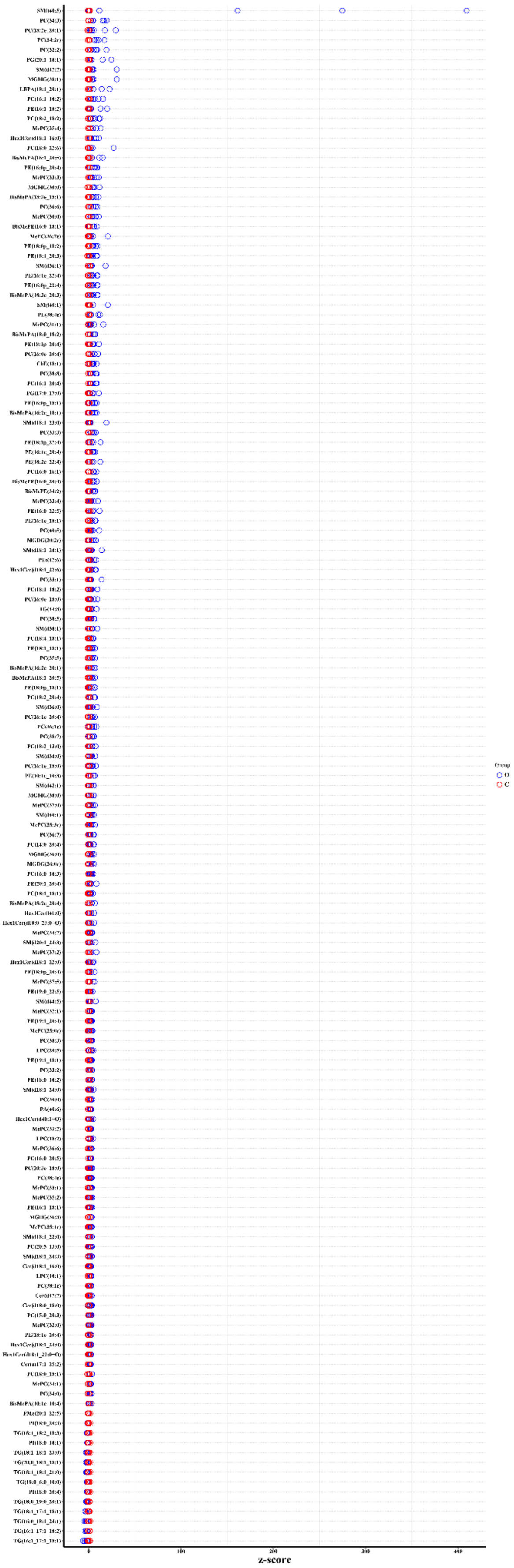

Figure S19 Liver lipidome Z score

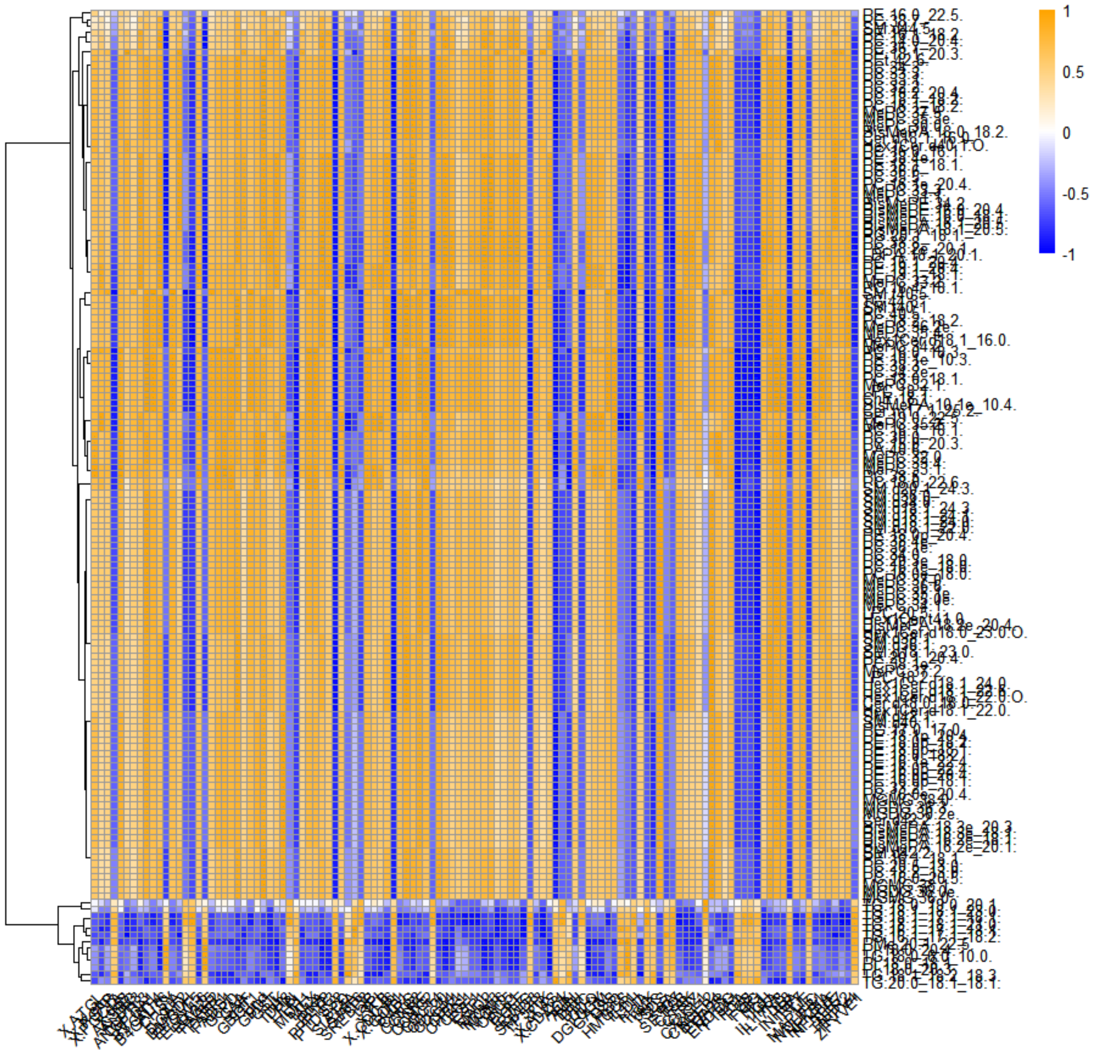

Figure S20 Correlation analysis between transcriptome and lipidome

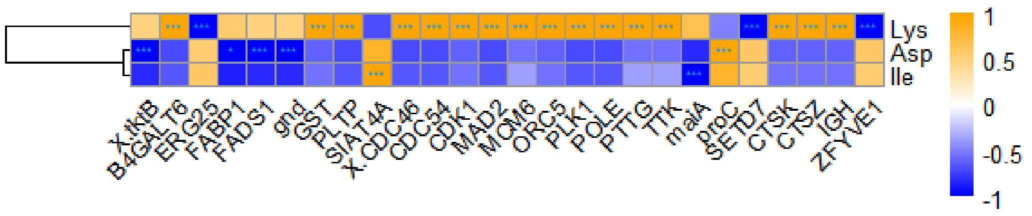

Figure S21 Correlation analysis between transcriptome and amino acids

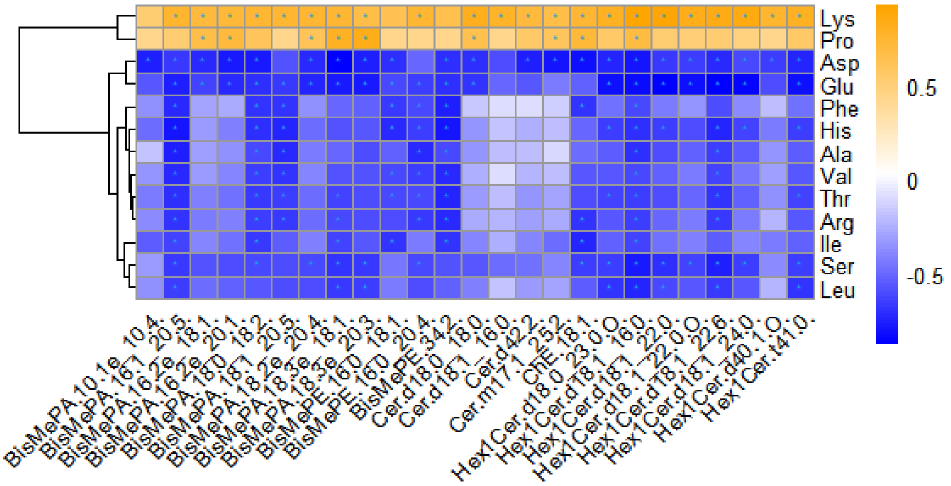

Figure S22 Correlation analysis between lipidome and amino acids-1

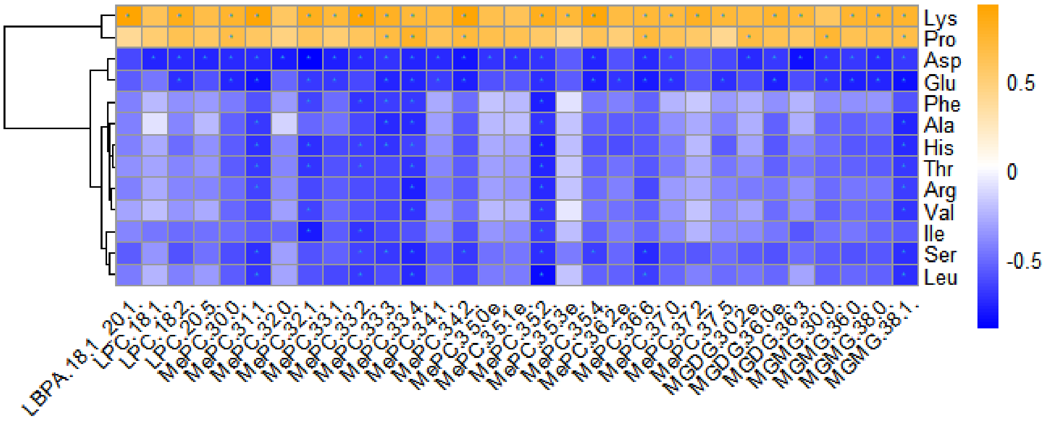

Figure S23 Correlation analysis between lipidome and amino acids-2

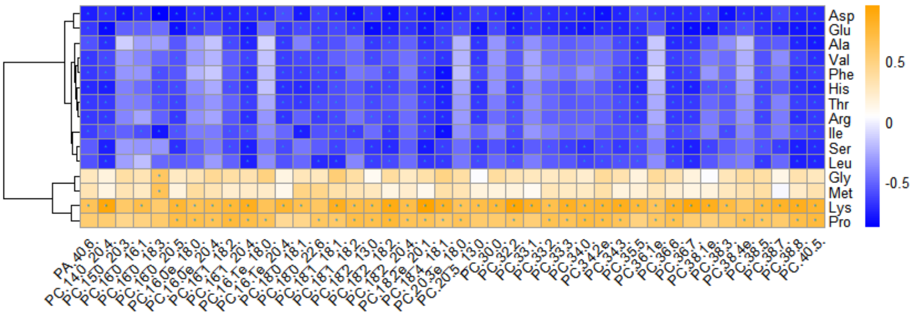

Figure S24 Correlation analysis between lipidome and amino acids-3

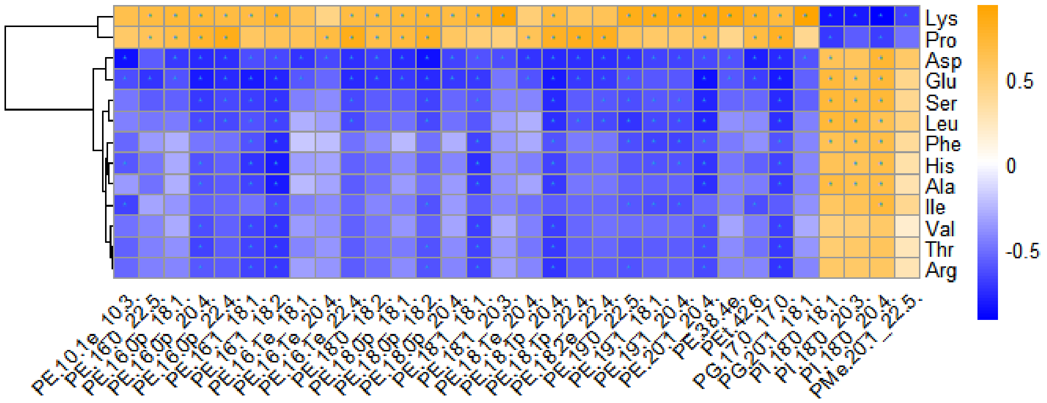

Figure S25 Correlation analysis between lipidome and amino acids-4

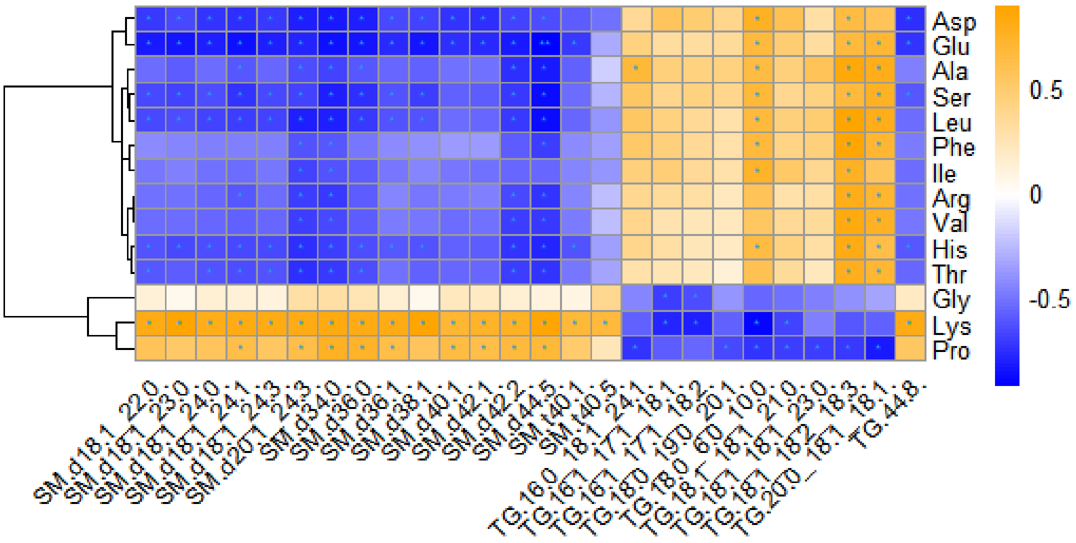

Figure S26 Correlation analysis between lipidome and amino acids-5

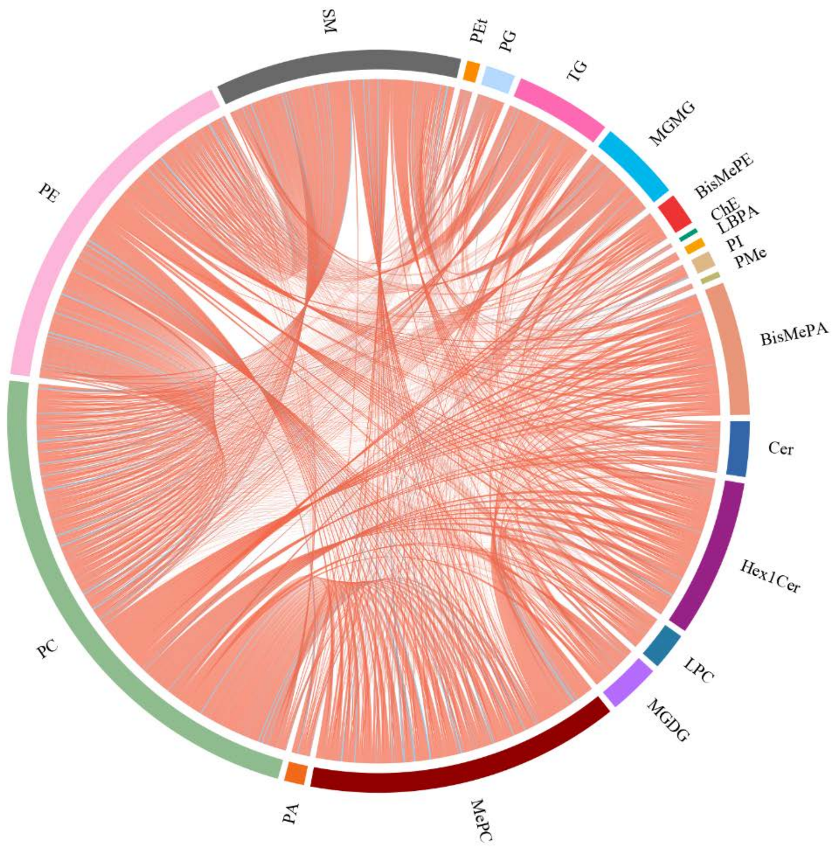

Figure S27 Correlation analysis between different lipids-chordal graph
