# Supplementary material for: Multi-omics reveals goose fatty liver formation from metabolic reprogramming
Source: Front Vet Sci. 2024 Jan 29;11:1122904. doi: 10.3389/fvets.2024.1122904 (PMC10859500; doi:10.3389/fvets.2024.1122904)
Supplement: Supplementary file 3 [file Data_Sheet_3.pdf]

Table S1 Sequencing data statistics

Sample		Reads No.	Bases (bp)	Q30 (bp)	N (%)	Q20 (%)	Q30 (%)
Liver	C1	50976130	7646419500	7242176898	0.000705	98.05	94.71
	C2	51091110	7663666500	7223979419	0.000794	97.77	94.26
	C3	55692024	8353803600	7889557586	0.0008	97.96	94.44
	O1	48605560	7290834000	6906209645	0.000716	98.03	94.72
	O2	47314650	7097197500	6664963606	0.000713	97.57	93.9
	O3	43270964	6490644600	6134159251	0.000704	97.89	94.5
SF	C1	60008852	9001327800	8107216789	0	95.6	90.06
	C2	77497496	11624624400	10364052548	0	94.87	89.15
	C3	69081710	10362256500	9315142323	0	95.47	89.89
	O1	43076038	6461405700	6025511261	0.000229	97.34	93.25
	O2	47458752	7118812800	6510964621	0.000226	96.27	91.46
	O3	45616476	6842471400	6336589695	0.000227	97.02	92.6
AF	C1	48941222	7341183300	6815935867	0.000232	97.05	92.84
	C2	49232690	7384903500	6851495299	0.000228	97.03	92.77
	C3	45910844	6886626600	6386473896	0.000225	96.97	92.73
	O1	44963772	6744565800	6300823521	0.000226	97.49	93.42
	O2	50631272	7594690800	7070352330	0.000227	97.31	93.09
	O3	52092022	7813803300	7247361390	0.000229	97.12	92.75
IF	C1	48040718	7206107700	6715324596	0.000787	97.06	93.18
	C2	47812368	7171855200	6695480394	0.000778	97.19	93.35
	C3	47019378	7052906700	6504998968	0.000228	96.65	92.23
	O1	50305188	7545778200	7087193716	0.000787	97.58	93.92
	O2	55297636	8294645400	7787289803	0.000786	97.56	93.88
	O3	52025224	7803783600	7348790411	0.000721	97.71	94.16

Note: Reads No.: Total number of reads.

Bases(bp): Total number of bases.

Q30 (bp): The total number of bases with a base identification accuracy of 99.9% or more.

N (%): Fuzzy base percentage.

Q20(%): Percentage of bases with a base identification accuracy of 99.9% or more.

Q30(%): Percentage of bases with a base identification accuracy of 99.9% or more.

C = Control group; O = Overfeeding group. SF = subcutaneous fat tissue. AF = abdominal fat tissue. IF = intestine-mesentery fat tissue.

Table S2 Filtering data

Sample		Clean Reads No.	Clean Data (bp)	Clean Reads %	Clean Data %
Liver	C1	47882616	7182392400	93.93	93.93
	C2	48057734	7208660100	94.06	94.06
	C3	52082742	7812411300	93.51	93.51
	O1	45690216	6853532400	94	94
	O2	44353576	6653036400	93.74	93.74
	O3	40644772	6096715800	93.93	93.93
SF	C1	56411048	8461657200	94	94
	C2	72271706	10840755900	93.25	93.25
	C3	64851870	9727780500	93.87	93.87
	O1	40099270	6014890500	93.08	93.08
	O2	43903182	6585477300	92.5	92.5
	O3	42325034	6348755100	92.78	92.78
AF	C1	45275908	6791386200	92.51	92.51
	C2	45632706	6844905900	92.68	92.68
	C3	42501094	6375164100	92.57	92.57
	O1	42066158	6309923700	93.55	93.55
	O2	47224864	7083729600	93.27	93.27
	O3	48572126	7285818900	93.24	93.24
IF	C1	44188168	6628225200	91.98	91.98
	C2	44017024	6602553600	92.06	92.06
	C3	42997378	6449606700	91.44	91.44
	O1	46886482	7032972300	93.2	93.2
	O2	51579272	7736890800	93.27	93.27
	O3	48558610	7283791500	93.33	93.33

Note: Clean Reads No: The number of high-quality reads.

Clean Data (bp): The number of high-quality bases.

Clean Reads %: Percentage of high-quality reads to sequenced reads.

Clean Data %: Percentage of high-quality sequence bases to sequenced bases.

C = Control group; O = Overfeeding group. SF = subcutaneous fat tissue. AF = abdominal fat tissue. IF = intestine-mesentery fat tissue.

Table S3 RNASeq Map statistics

Sample		Clean_ Reads	Total_ Mapped	Multiple_ Mapped	Uniquely_ Mapped
Liver	C1	47882616	38198811 (79.78%)	1144807 (3.00%)	37054004 (97.00%)
	C2	48057734	36088123 (75.09%)	1130376 (3.13%)	34957747 (96.87%)
	C3	52082742	43170008 (82.89%)	1324533 (3.07%)	41845475 (96.93%)
	O1	44353576	30730651 (69.29%)	786695 (2.56%)	29943956 (97.44%)
	O2	45690216	34840558 (76.25%)	948712 (2.72%)	33891846 (97.28%)
	O3	40644772	28708112 (70.63%)	760290 (2.65%)	27947822 (97.35%)
SF	C1	56411048	50165987 (88.93%)	1354683 (2.70%)	48811304 (97.30%)
	C2	72271706	61511039 (85.11%)	519489 (0.84%)	60991550 (99.16%)
	C3	64851870	56785048 (87.56%)	1243560 (2.19%)	55541488 (97.81%)
	O1	40099270	33216279 (82.84%)	682416 (2.05%)	32533863 (97.95%)
	O2	43903182	31714634 (72.24%)	621737 (1.96%)	31092897 (98.04%)
	O3	42325034	34493072 (81.50%)	726840 (2.11%)	33766232 (97.89%)
AF	C1	45275908	35939428 (79.38%)	754080 (2.10%)	35185348 (97.90%)
	C2	45632706	34449801 (75.49%)	736318 (2.14%)	33713483 (97.86%)
	C3	42501094	33285729 (78.32%)	607351 (1.82%)	32678378 (98.18%)
	O1	42066158	35160735 (83.58%)	782142 (2.22%)	34378593 (97.78%)
	O2	47224864	39862901 (84.41%)	823964 (2.07%)	39038937 (97.93%)
	O3	48572126	39337427 (80.99%)	844725 (2.15%)	38492702 (97.85%)
IF	C1	44188168	30406789 (68.81%)	693845 (2.28%)	29712944 (97.72%)
	C2	44017024	33907437 (77.03%)	820468 (2.42%)	33086969 (97.58%)
	C3	42997378	28721771 (66.80%)	716496 (2.49%)	28005275 (97.51%)
	O1	46886482	38347209 (81.79%)	929302 (2.42%)	37417907 (97.58%)
	O2	51579272	41511016 (80.48%)	894598 (2.16%)	40616418 (97.84%)
	O3	48558610	39645479 (81.64%)	943868 (2.38%)	38701611 (97.62%)

Note: Clean Reads: Total Mapped.

Total Mapped: Total Mapped / Clean Reads.

Multiple Mapped: Multiple Mapped / Total Mapped.

Uniquely Mapped: Uniquely Mapped / Total Mapped.

C = Control group; O = Overfeeding group. SF = subcutaneous fat tissue. AF = abdominal fat tissue. IF = intestine-mesentery fat tissue.

Table S4 DEGs involved in glucolipid metabolism, cell cycle, amino acids metabolism and inflammatory response in goose liver

pathway	gene	up/ down	pathway	gene	up/ down	pathway	gene	up/ down	pathway	gene	up/ down
1	HK	up	1	IDH3	up	2	CDC46	up	3	maiA	up
1	ANGPTL3	up	1	AGPAT1_2	up	2	CDC45	up	3	GATM	up
1	eno	up	1	B3GNT4	up	2	BUB1	up	3	DGLUCY	up
1	APOA1	up	1	PPP1R3	up	2	CAPG	up	3	HMGCS	down
1	pfkA	up	1	ELOVL6	up	2	AGPAT6	up	3	ldh	down
1	ALDH3	up	1	B4GALT6	up	2	CCNE	up	3	IL4I1	down
1	ACSBG	up	1	GPD1	up	2	GAA	up	3	proC	down
1	talB	up	1	LPL	down	2	CDC54	up	3	serA	down
1	DGAT2	up	1	ELOVL5	down	2	MCM3	up	3	DMGDH	down
1	PGD	up	1	LPIN	down	2	CCNB2	up	3	AUH	down
1	FAXDC2	up	1	SREBP2	down	2	MAD2	up	3	SETD7	down
1	galF	up	1	PTDSS2	down	2	MCM6	up	3	metK	down
1	FAAH2	up	1	GNA13	down	2	ORC5	up	3	AOX	down
1	tktB	up	1	ERG25	down	2	CHEK1	up	3	DDC	down
1	MDH1	up	1	SIAT4A	down	2	E2F1	up	4	ENDOG	up
1	ELOVL6	up	1	CHSY	down	2	COP1	down	4	F13A1	up
1	gst	up	1	SIAT8E	down	2	SMAD3	down	4	CTSB	up
1	tpiA	up	2	CKS1	up	2	CDC14	down	4	CD120b	up
1	FADS1	up	2	POLE	up	3	ALAS	up	4	TRPV4	up
1	G6PD	up	2	CCNB3	up	3	DDO	up	4	MAP1LC	down
1	MLX	up	2	CDC20	up	3	HGD	up	4	CFH	down
1	xylB	up	2	PTTG	up	3	DBH	up	4	ZFYVE1	down
1	ATGL	up	2	CDK1	up	3	MIF	up	/	/	/
1	GBGT1	up	2	MPS1	up	3	gltA	up	/	/	/
1	SCD	up	2	TP53I3	up	3	SDS	up	/	/	/

Note: DEGs come from goose liver transcriptome sequencing (control group *vs* overfeeding group) (n=3). “1” represents glucolipid metabolism, “2” represents cell cycle, “3” represents amino acids metabolism, “4” represents inflammatory response.

Table S5 DEGs involved in glucolipid metabolism, cell cycle, amino acids metabolism and inflammatory response in goose subcutaneous fat tissue

path way	gene	up/ down	path way	gene	up/ down	path way	gene	up/ down	path way	gene	up/ down	path way	gene	up/ down
1	LASS2_4	up	1	TPI	up	1	AS160	down	1	UGCG	down	4	TXNIP	up
1	STARD3	up	1	galE	up	1	MTP	down	1	GYS	down	4	MON1	up
1	PFKFB2	up	1	rpe	up	1	APOB	down	1	FASN	down	4	VPS16	up
1	DPPL	up	1	gmd	up	1	NCEH1	down	1	GPAT1_2	down	4	SERPINB2	up
1	CHK	up	1	fumC	up	1	PSAP	down	1	CHAT	down	4	ACTR2	up
1	ECI1	up	1	ACLY	up	1	ASAH2	down	1	glpA	down	4	DAPK	up
1	SMPD2	up	1	gltA	up	1	MLX	down	1	GPD1	down	4	BECN	up
1	ASAH1	up	1	ALDO	up	1	EBF, COE	down	2	THG1	up	4	MLST8	up
1	GAA	up	1	NAGA	up	1	PPARA	down	2	RFC3_5	up	4	TNFRSF21	up
1	ACTA2	up	1	GBA	up	1	PPP1R3	down	2	RPA3	up	4	IFNGR2	up
1	ELOVL1	up	1	MAN2C1	up	1	PTPN1	down	2	RFA2	up	4	TFPI	up
1	MLX	up	1	pyk	up	1	ABCG8	down	2	ZBTB17	up	4	CD141	up
1	B3GNT4	up	1	pfkA	up	1	ABCG5	down	2	CCND3	up	4	F8	up
1	B4GALT4	up	1	galK	up	1	ABCC1	down	2	EI24	up	4	EIF5	up
1	B4GALT2	up	1	FDFT1	up	1	ABCA1	down	2	CDC20	up	4	SKP1	up
1	HPSE2	up	1	LCAT	up	1	PRKAR	down	2	APC2	up	4	MCL1	up
1	PIGX	up	1	GNPAT	up	1	BK	down	2	MAD2	up	4	UVRAG	down
1	HADHA	up	1	fabD	up	1	B3GALT5	down	2	CHEK1	up	4	VPS3	down
1	NRBF1	up	1	GPAT1_2	up	1	SIAT7C	down	2	MCM5	up	4	MUC5B	down
1	ECHS1	up	1	aceF	up	1	SIAT9	down	2	CDK1	up	4	CARD11	down
1	ACAA2	up	1	atoB	up	1	SIAT8E	down	2	DYRK2_3_4	down	4	ITK	down
1	FLOT	up	1	DLD	up	1	DPL1	down	2	ZC3H12	down	4	LCP2	down
1	PPP1R3	up	1	bcd	up	1	GPLD1	down	2	SESN1_3	down	4	IL1R2	down
1	PIGT	up	1	SDHD	up	1	ENPP2	down	2	ZMAT3	down	4	C6	down
1	PIGS	up	1	SC5DL	up	1	PLD1_2	down	2	PERP	down	4	C5	down
1	PIGW	up	1	OGDH	up	1	PTEN	down	2	SMC2	down	4	C3	down
1	GPI2	up	1	gapA	up	1	INPP4	down	2	SCC2	down	4	C4	down
1	CHPF2	up	1	ADH5	up	1	PFKFB3	down	2	SCC3	down	4	SERPINF2	down
1	SIAT7F	up	1	G6PD	up	1	gnl, RGN	down	2	SCC1	down	4	F13A1	down
1	ACATN	up	1	PGD	up	1	LIPA	down	2	CDC14	down	4	SERPIND1	down
1	fcl	up	1	IDH3	up	1	cdsA	down	2	SMC1	down	4	SERPINC1	down
1	gpmA	up	1	MDH2	up	1	prsA	down	2	WEE1	down	4	A2M	down
1	manA	up	1	LASS2_4	down	1	adk	down	2	ORC2	down	4	PROS1	down
1	rpiA	up	1	ɅTMR6_7_	down	1	PIP4K2	down	4	S100A9	up	4	TRAF6	down

Note: DEGs come from goose subcutaneous fat tissue transcriptome sequencing (control group *vs* overfeeding group) (n=3). “1” represents glucolipid metabolism, “2” represents cell cycle, “3” represents amino acids metabolism, “4” represents inflammatory response.

Table S6 DEGs involved in glucolipid metabolism, cell cycle, amino acids metabolism and inflammatory response in goose abdominal fat tissue

pathway	gene	up/ down	pathway	gene	up/ down	pathway	gene	up/ down	pathway	gene	up/ down
1	G6PC	down	1	LIPA	up	1	LPL	up	1	SGPP1	up
1	UGT	down	1	pyk	up	1	MGLL	up	1	CPLA2	up
1	PLB	down	1	GPI	up	1	SGP1	up	1	ENO, eno	up
1	PLD3_4	down	1	AGPAT3_4	up	1	B4GALT6	up	1	LMNB	up
1	TSC2	down	1	CTSD	up	1	CHK	up	1	UGT8	up
1	ALDH3	down	1	SGMS	up	1	pgk	up	1	PLA1A	up
1	LPAR6	down	1	MGAT2	up	1	GPAT1_2	up	2	CCNE	down
1	IP6K	down	1	ldh	up	1	B3GALT2	up	2	FUK	down
1	PAFAH	down	1	LPCAT1_2	up	1	LASS2_4	up	2	CAPD2	down
1	ACACB	down	1	AGPAT5	up	1	DGAT2	up	2	YWHAG_H	up
1	CPLA2	down	1	psd	up	1	MINPP1	up	4	PAI2	up
1	LRP1	up	1	SPT	up	1	CD141	up	4	TRPA1	up
1	GALC	up	1	DPL1	up	1	fadD	up	4	CD107	up
1	B3GNT5	up	1	ADPGK	up	1	fabF	up	4	F13A1	up

Note: DEGs come from goose abdominal fat tissue transcriptome sequencing (control group *vs* overfeeding group) (n=3). "1" represents glucolipid metabolism, "2" represents cell cycle, "3" represents amino acids metabolism, "4" represents inflammatory response.

Table S7 DEGs involved in glucolipid metabolism, cell cycle, amino acids metabolism and inflammatory response in goose intestine- mesentery fat tissue

pathway	gene	up/ down	pathway	gene	up/ down	pathway	gene	up/ down	pathway	gene	up/ down
1	ENPP2	down	1	CTSD	up	1	UGDH	up	2	SESN1_3	up
1	DCXR	down	1	B4GALT6	up	1	SPT	up	4	CFH	down
1	IP6K	down	1	fadD	up	1	ELOVL5	up	4	H2A	down
1	ALDH	down	1	pyk	up	1	DHCR24	up	4	MLKL	down
1	fabD	down	1	CPT1A	up	1	NAGA	up	4	C8G	down
1	FUK	down	1	LPCAT1_2	up	1	DHCR7	up	4	PROC	down
1	GPAT1_2	down	1	pgk	up	1	AKR1A1	up	4	C1QG	up
1	G6PC	down	1	ACER3	up	1	LCAT	up	4	CD107	up
1	ECHS1	down	1	ADPGK	up	1	PIGK	up	4	F13A1	up
1	PLD3_4	down	1	AGPAT5	up	1	PPP1R3	up	4	TRPA1	up
1	TSC1	down	1	GALC	up	1	galE	up	4	C1QB	up
1	ACACB	down	1	HACD	up	1	SGPL1	up	4	VSIG4	up
1	NTE	down	1	CHK	up	1	GRM7	up	4	PAI2	up
1	PIP5K	down	1	ENPP6	up	1	DGAT2	up	4	CASP10	up
1	atoB	down	1	MINPP1	up	1	GPI1	up	4	F3	up
1	PLB1	down	1	AGPAT3_4	up	2	CCNE	down	4	TLR3	up
1	LPL	up	1	ETNK, EKI	up	2	SESN1_3	down	4	PLAU	up
1	LIPA	up	1	psd	up	2	KAI1	up	4	LMNB	up
1	PSAP	up	1	GPAT1_2	up	2	fabF	up	4	CD141	up
1	MESO1	up	1	gpmA	up	2	PPP2R2	up	/	/	/
1	LASS2_4	up	1	SIAT7F	up	2	SCOTIN	up	/	/	/

Note: DEGs come from goose intestine-mesentery fat tissue transcriptome sequencing (control group *vs* overfeeding group) (n=3). “1” represents glucolipid metabolism, “2” represents cell cycle, “3” represents amino acids metabolism, “4” represents inflammatory response.
